# Caveolin‐1 deficiency induces premature senescence with mitochondrial dysfunction

**DOI:** 10.1111/acel.12606

**Published:** 2017-05-17

**Authors:** Dong‐Min Yu, Seung Hee Jung, Hyoung‐Tae An, Sungsoo Lee, Jin Hong, Jun Sub Park, Hyun Lee, Hwayeon Lee, Myeong‐Suk Bahn, Hyung Chul Lee, Na‐Kyung Han, Jesang Ko, Jae‐Seon Lee, Young‐Gyu Ko

**Affiliations:** ^1^ Tunneling Nanotube Research Center Korea University Seoul 02841 Korea; ^2^ Division of Life Sciences Korea University Seoul 02841 Korea; ^3^ Department of Molecular Medicine Inha University College of Medicine Incheon 22212 Korea; ^4^ Hypoxia‐related Disease Research Center Inha University College of Medicine Incheon 22212 Korea

**Keywords:** cardiolipin, caveolin‐1, mitochondria, senescence, SIRT1

## Abstract

Paradoxical observations have been made regarding the role of caveolin‐1 (Cav‐1) during cellular senescence. For example, caveolin‐1 deficiency prevents reactive oxygen species‐induced cellular senescence despite mitochondrial dysfunction, which leads to senescence. To resolve this paradox, we re‐addressed the role of caveolin‐1 in cellular senescence in human diploid fibroblasts, A549, HCT116, and Cav‐1^*−/−*^ mouse embryonic fibroblasts. Cav‐1 deficiency (knockout or knockdown) induced cellular senescence via a p53‐p21‐dependent pathway, downregulating the expression level of the cardiolipin biosynthesis enzymes and then reducing the content of cardiolipin, a critical lipid for mitochondrial respiration. Our results showed that Cav‐1 deficiency decreased mitochondrial respiration, reduced the activity of oxidative phosphorylation complex I (CI), inactivated SIRT1, and decreased the NAD ^+^/NADH ratio. From these results, we concluded that Cav‐1 deficiency induces premature senescence via mitochondrial dysfunction and silent information regulator 2 homologue 1 (SIRT1) inactivation.

## Introduction

Cellular senescence is defined as an irreversible cellular growth arrest. Human fibroblasts have a maximum division number before undergoing senescence (Hayflick & Moorhead, 1961). Cellular senescence is prematurely induced by diverse stresses, such as oxidative stress, oncogene activation, and genomic instability (Campisi & d'Adda di Fagagna, 2007; Lee & Lee, 2014). Senescent cells have various phenotypes, including fried egg‐like morphology, β‐galactosidase (β‐Gal) activation, heterochromatin focus formation, and altered gene expression and protein processing (Shay & Roninson, 2004; Byun *et al*., 2015). The tumor protein p53 is an essential senescence‐inducing protein because its overexpression induces, but its disruption prevents, cellular senescence (Sugrue *et al*., 1997; Ben‐Porath & Weinberg, 2005). The p53 protein is deacetylated and inactivated by silent information regulator 2 homologue 1 (SIRT1), an NAD‐dependent deacetylase enzyme (Vaziri *et al*., 2001). Thus, SIRT1 activation prevents cellular senescence, whereas its inactivation increases the transcriptional activity of p53 (Tang *et al*., 2008; Wang *et al*., 2012). These findings indicate that the SIRT1‐p53 pathway is critical for regulating cellular senescence.

Caveolae, omega‐shaped plasma membrane invaginations, play important roles in enriching and organizing signaling molecules and clathrin‐independent endocytosis (Parat, 2009). Because caveolin‐1 (Cav‐1) is predominantly found in the caveolae and Cav‐1 deficiency prevents caveolae formation, Cav‐1 is a critical structural component in caveolar membranes (Parat, 2009). Cav‐1 functions as a scaffolding protein, regulating different signal transduction pathways and interacting with various molecules, such as cholesterol, the epidermal growth factor (EGF) receptor, heterotrimeric G‐proteins, endothelial nitric oxide synthase (eNOS), Src, and H‐Ras (Li *et al*., 1996; Parat, 2009).

Cav‐1 is upregulated during replicative and premature senescence. For example, Cav‐1 is upregulated in senescent HDFs, mesenchymal stem cells, and bone marrow stromal cells (Park *et al*., 2000; Sun *et al*., 2009). Consistent with the results from senescent cells, Cav‐1 expression is highly increased in the brain, spleen, and lung of old rats (Park *et al*., 2000; Kang *et al*., 2006). The Cav‐1 expression level is also highly elevated after exposure to hydrogen peroxide in NIH‐3T3 cells (Volonte *et al*., 2002). Because Cav‐1 knockdown or knockout prevents stressor‐induced premature senescence in NIH‐3T3 cells and mouse embryonic fibroblasts (MEFs), Cav‐1 might be required for premature senescence (Volonte *et al*., 2002; Bartholomew *et al*., 2009). However, the requirement of Cav‐1 in premature senescence is challenged by other observations. Cav‐1 knockdown does not alter hydrogen peroxide‐induced premature senescence in HDFs, induces a senescence‐like morphological change, including increased cell size and formation of stress fibers in human endothelial cells, and prevents *in vitro* and *in vivo* cellular growth in human colorectal cancer cells (Chretien *et al*., 2008; Ha *et al*., 2012; Madaro *et al*., 2013). Cav‐1 knockout mice have aging‐related phenotypes in various organs. Cav‐1 knockout mice exhibit neurodegeneration with premature aging, reduced capacity of liver regeneration, fat atrophy, and pathological hypertrophy of heart and insulin resistance (Cohen *et al*., 2003; Head *et al*., 2010; Briand *et al*., 2011; Trajkovski *et al*., 2011). In MEFs and mouse liver, Cav‐1 knockout also leads to mitochondrial dysfunction, which is a strong inducer of premature senescence (Bosch *et al*., 2011; Asterholm *et al*., 2012).

To resolve the conflicting observations for the role of Cav‐1 in premature senescence, we re‐addressed whether Cav‐1 is required for cellular senescence in different cell lines. We demonstrated that Cav‐1 deficiency induced cellular senescence by activating a p53‐p21 pathway. Interestingly, Cav‐1 deficiency decreased cardiolipin level, a critical component for oxidative phosphorylation (OXPHOS) functions. Finally, we showed that Cav‐1 deficiency induced cellular senescence with impaired mitochondrial respiration and SIRT1 inactivation.

## Results

### Caveolin‐1 knockdown induces premature senescence

**Figure 1:**
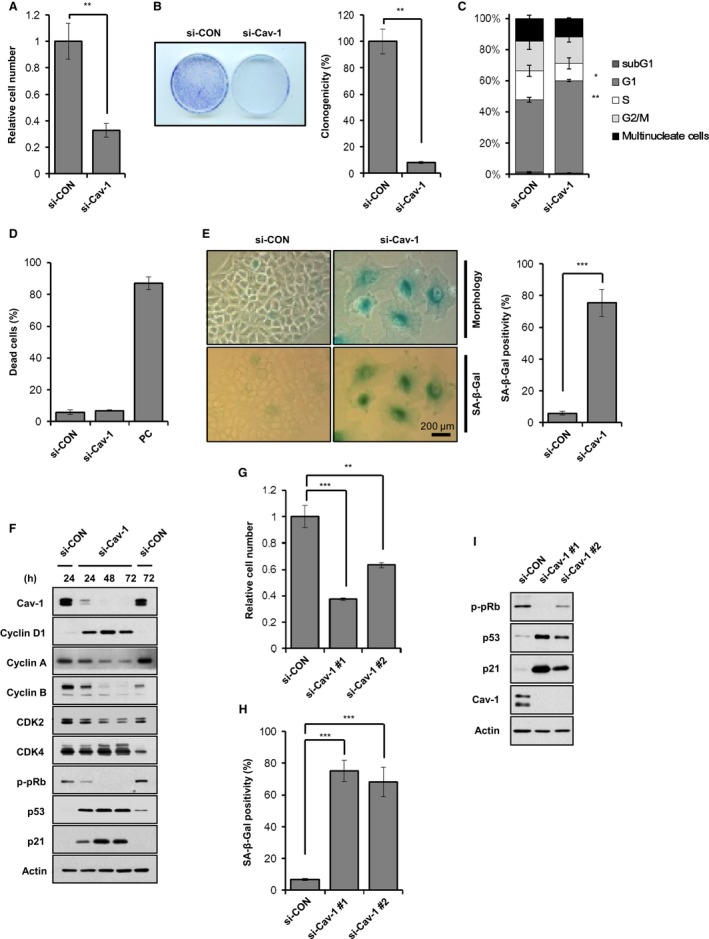
Caveolin‐1 knockdown induces premature senescence. A549 cells were treated with 100 nm si‐control (si‐CON) or si‐Cav‐1 (A–F). Three days after siRNA treatment, the cell were counted and presented as relative values (A). Seven days after siRNA treatment, the colonies were counted in a colony‐forming assay and presented as relative values (B). Three days after siRNA treatment, cell cycle distributions were analyzed by FACS (C). Three days after siRNA treatment, the dead cell population was counted after Trypan blue staining. We used 10 μg mL^−1^ doxorubicin‐treated A549 cells as a positive control (PC) (D). Five days after siRNA treatment, cellular morphology and β‐gal staining positivity were observed. β‐gal positivity was statistically quantified by calculating the ratio of stained cells to total cells (E). Cells were harvested at the indicated times after siRNA treatment and were subjected to immunoblotting analysis (F). A549 cells were treated with si‐CON or two different si‐Cav‐1 for 3 days (G–I). Relative cell number (G) and β‐Gal staining positivity (H) were quantified, and immunoblotting was performed (I). All data are shown as the mean ± standard deviations (SD). Statistical significance was determined using Student's *t*‐test. *P <* *0.05, **P <* *0.01, and ***P <* *0.001.

To address whether Cav‐1 is necessary for premature senescence, we first monitored cellular proliferation by counting cell numbers and using a colony‐forming assay after Cav‐1 knockdown in A549 human lung carcinoma cells, which highly express Cav‐1. Cav‐1 knockdown significantly decreased cellular proliferation and colony‐forming capacity compared with the si‐control‐treated cells (Fig. 1). In the cell cycle analysis by fluorescence‐activated cell sorting (FACS), Cav‐1 knockdown increased the G_1_ phase cell population but decreased the S phase cell population without changing the sub‐G_1_ phase cell population (Fig. 1C). In addition, Cav‐1 knockdown did not increase Trypan blue staining (Fig. 1D). Unexpectedly, Cav‐1 knockdown increased β‐gal positivity and changed the cellular morphology from a normal epithelial shape to a fried egg‐like shape (Fig. 1E). From these results, we concluded that Cav‐1 knockdown induces cellular senescence but not cell death in A549 cells.

Next, we assessed the expression changes of cell cycle regulatory proteins after Cav‐1 knockdown in A549 cells. Cyclin D1 expression was elevated, whereas pRb phosphorylation and cyclin A and B expression were decreased after Cav‐1 knockdown (Fig. 1F). These data confirmed what we observed in Fig. 1C, where Cav‐1 knockdown arrested the cell cycle in the G_1_ phase. Two other G_1_ arrest markers, p53 and p21, were also significantly elevated by Cav‐1 knockdown. To exclude any off‐target effects of Cav‐1 siRNA, we used two different Cav‐1 siRNAs in A549 cells and observed the senescence phenotypes. As determined by cell proliferation, β‐gal staining positivity, and p‐pRb, p53, and p21 expression levels (Fig. 1G–I), both Cav‐1 siRNAs induced cellular senescence. Moreover, introduction of RNAi‐resistant Cav‐1 in which RNAi‐targeting DNA sequence has silent mutations and therefore was not knockdowned by Cav‐1 siRNA was able to rescue Cav‐1 knockdown‐induced cellular senescence (Fig. S1, Supporting information). These results confirmed that Cav‐1 deficiency‐induced cellular senescence was indeed caveolin‐1 specific.

To generalize Cav‐1 knockdown‐induced cellular senescence, we repeated this set of experiments in HCT116 human colorectal carcinoma cells, human diploid fibroblasts (HDFs), and H460 human lung cancer cells. As determined by cellular proliferation, β‐gal staining positivity and p‐pRb, p53, and p21 expression levels (Fig. S2, Supporting information), Cav‐1 knockdown induced cellular senescence in all cell lines we tested. From these results, we concluded that Cav‐1 knockdown induces premature senescence in normal and cancer cells.

### Caveolin‐1 knockdown induces premature senescence via a p53‐p21 pathway

**Figure 2:**
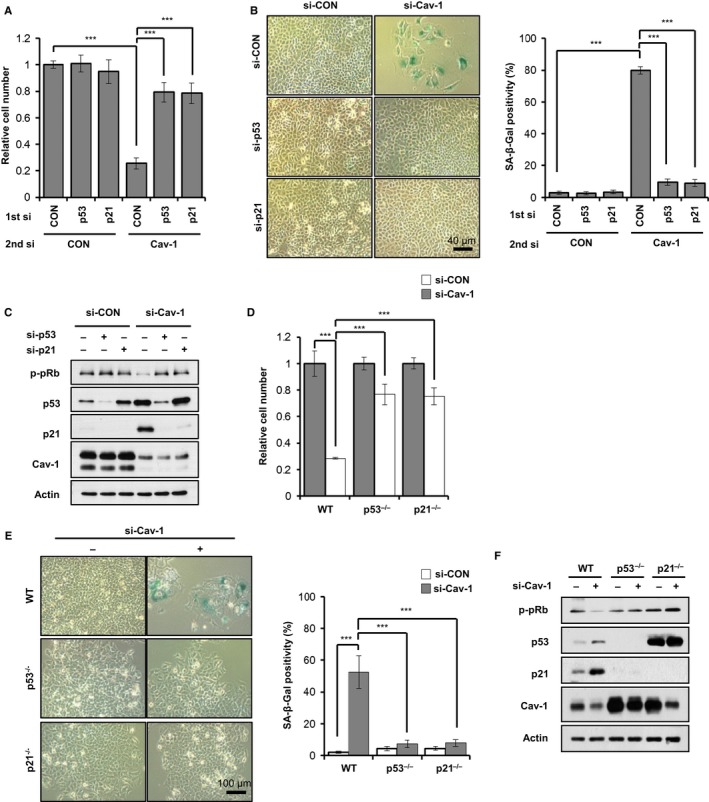
Cav‐1 knockdown induces premature senescence via a p53‐p21 pathway. A549 cells were treated with si‐CON, si‐p53, or si‐p21 (1^st^ si). After 24 h, the A549 cells were further treated with si‐CON or si‐Cav‐1 (2^nd^ si) (A–D). Quantification of relative cell number (A), β‐Gal staining positivity (B), and immunoblotting (C) were determined 3 days after the second transfection. HCT116 wild‐type, p53^*−*/*−*^ and p21^*−*/*−*^ cells were transfected with si‐CON or si‐Cav‐1 (D–F). Relative cell number (D) and β‐Gal staining positivity (E) were quantified, and immunoblotting was performed (F) 3 days after the second transfection. All data are shown as the mean ± SD. Statistical significance was determined using Student's *t*‐test. *P <* *0.05, **P <* *0.01, and ***P <* *0.001.

To investigate whether p53 and p21 were essential for Cav‐1 knockdown‐induced cellular senescence, we monitored cellular senescence in A549 cells after double knockdowns of Cav‐1 and p53 or Cav‐1 and p21. Analyses of cell numbers, β‐gal staining positivity, and p‐pRb, p53, and p21 expression levels (Fig. 2A–C) showed that p53 or p21 knockdown prevented Cav‐1 knockdown‐induced cellular senescence. To further confirm the involvement of p53 or p21 in Cav‐1 knockdown‐induced cellular senescence, we observed cellular senescence after Cav‐1 knockdown in p53‐ or p21‐null HCT116 cells. Cav‐1 knockdown induced cellular senescence in HCT116 wild‐type cells but not in HCT116 p53^*−*/*−*^ and p21^*−*/*−*^ cells (Fig. 2D–F), indicating that a p53‐p21 pathway is necessary for Cav‐1‐deficiency‐induced cellular senescence.

### Caveolin‐1 knockdown induces mitochondrial dysfunction

**Figure 3:**
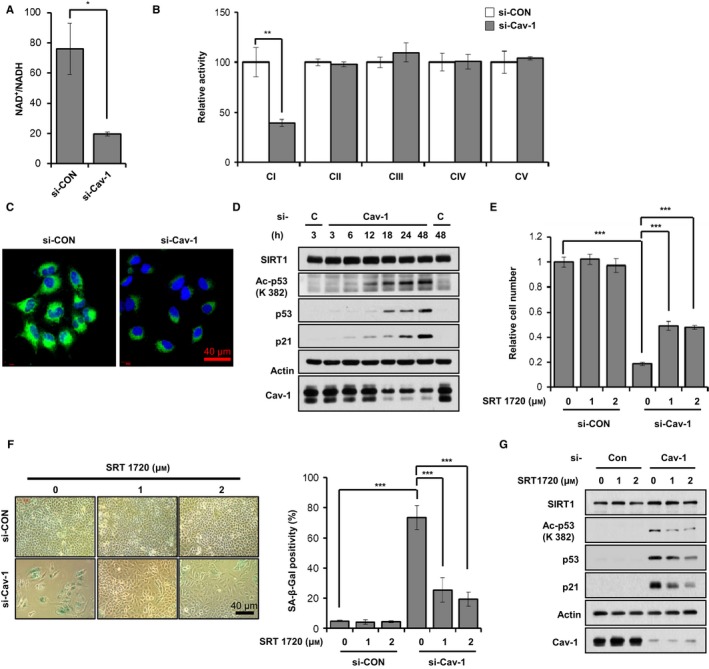
Caveolin‐1 knockdown leads to mitochondrial dysfunction and SIRT1 inactivation. A549 cells were treated with 100 nM si‐CON or si‐Cav‐1 for 24 h (A–C). Intracellular NAD ^+^/NADH ratio was measured using a quantitation colorimetric assay kit (A). CI‐CV activity levels were analyzed as described in the Experimental procedure (B). The cells were stained with 100 nm NAO for 30 min (C). A549 cells were treated with 100 nm si‐CON or si‐Cav‐1 for the indicated times. Whole cell lysates were determined by immunoblotting for SIRT1, acetylated p53 (Ac‐p53), p53, p21, and Cav‐1 using actin as a loading control (D). A549 cells were treated with 100 nm si‐CON or si‐Cav‐1 for 6 h and then treated with 1 or 2 μm SRT1720 for 3 days (E–G). Relative cell number (E) and β‐Gal staining positivity (F) were quantified, and immunoblotting was performed (G). All data are shown as the mean ± SD. Statistical significance was determined using Student's *t*‐test. *P <* *0.05, **P <* *0.01 and ***P <* *0.001.

Because mitochondrial dysfunction has been observed in the liver and in MEFs obtained from Cav‐1^*−*/*−*^ mice, we also evaluated mitochondrial function after Cav‐1 knockdown in A549 cells. Cav‐1 knockdown significantly decreased the oxygen consumption rate (OCR), the extracellular acidification rate (ECAR), and the intracellular ATP level without changing the reactive oxygen species levels (Fig. S3A–D, Supporting information). In addition, the NAD^+^/NADH ratio was reduced to approximately 25% after Cav‐1 knockdown (Fig. 3A). These data indicate that Cav‐1 knockdown prevents mitochondrial respiration and ATP production without ROS generation. To explore the molecular mechanism of how Cav‐1 knockdown induces mitochondrial dysfunction, we evaluated the mitochondrial DNA (mtDNA) content and the expression levels of OXPHOS complex subunits. Neither the mtDNA content nor the expression levels of OXPHOS complex subunits were changed by Cav‐1 knockdown (Fig. S3E,F, Supporting information). Next, we measured the enzymatic activity of OXPHOS complexes I‐V (CI‐CV) after Cav‐1 knockdown. CI activity was reduced to approximately 40% of si‐control, whereas the activity levels of the other complexes (CII‐CV) were not changed by Cav‐1 knockdown (Fig. 3B). Because CI activity is regulated by cardiolipin, a major lipid of the inner mitochondrial membrane, we measured cardiolipin levels by staining cells with 10‐N‐nonyl acridine orange (NAO), a fluorescent cardiolipin indicator. NAO staining showed the significant decrease in cardiolipin in cells after Cav‐1 knockdown (Fig. 3C). To determine whether the decreased cardiolipin level was due to the reduction in cardiolipin biosynthesis or the increase in its turnover after Cav‐1 knockdown, we measured the cardiolipin level after radioactive labeling of phospholipid. After Cav‐1 knockdown cardiolipin level was decreased to less than 40% of si‐control‐treated cells after 24 h of labeling (Fig. S3H, Supporting information). We did not find a significant difference in cardiolipin turnover between cells treated with si‐control and cells treated with si‐Cav‐1 after 24 h of chase period (Fig. S3H, Supporting information). These data indicate that the decreased cardiolipin level after Cav‐1 knockdown was due to the decreased cardiolipin biosynthesis, not due to the increased cardiolipin turnover rate. It is imperative to determine whether the decreased cardiolipin biosynthesis alone could induce cellular senescence. Knocking down CDP‐diacylglycerol synthase 1 (CDS1) which is the first enzyme in cardiolipin biosynthesis pathway with two different siRNAs‐induced cellular senescence in A549 cells, as determined by cell numbers, β‐gal staining positivity, and p53 and p21 expression levels (Fig. S3I–K, Supporting information). These data demonstrate that Cav‐1 knockdown induces mitochondrial dysfunction by preventing cardiolipin biosynthesis.

Because Cav‐1 knockdown led to reduced mitochondrial CI activity (Fig. 3B), we hypothesized that Cav‐1 knockdown‐induced cellular senescence might result from CI dysfunction. To address this possibility, we investigated cellular senescence in A549 cells after the knockdown of NADH dehydrogenase (ubiquinone) flavoprotein 1 (NDUFV1), the first CI subunit that accepts electrons from NADH. CI subunit knockdown induced cellular senescence as determined by cell number counting, β‐staining positivity and p53, acetylated p53 and p21 expression levels (Fig. S4A–C, Supporting information), and reduced the OCR, ECAR, intracellular ATP levels and NAD^+^/NADH ratio without changing the ROS level as seen in Cav‐1 knockdown (Fig. S4D–H, Supporting information).

### SIRT1 inactivation is involved in Cav‐1 knockdown‐induced cellular senescence

Because Cav‐1 knockdown reduced the NAD^+^/NADH ratio, we speculated that SIRT1 is involved in Cav‐1 knockdown‐induced cellular senescence. Therefore, we measured SIRT1 activity by measuring acetylated p53 expression after Cav‐1 knockdown in A549 cells using immunoblotting. The acetylation of p53 was gradually increased with time after Cav‐1 knockdown (Fig. 3D). To further confirm SIRT1 inactivation by Cav‐1 knockdown, we treated Cav‐1 knockdown A549 cells with a SIRT1 activator, SRT1720, and observed reduced cellular senescence. Acetylation of p53 was decreased by SRT1720 treatment in Cav‐1 knockdown cells (Fig. 3G). As determined by cell numbers and β‐gal staining positivity (Fig. 3E,F), Cav‐1 knockdown‐induced cellular senescence was partially prevented in the presence of SRT1720. Next, we treated Cav‐1 knockdown A549 cells with another SIRT1 activator, nicotinamide mononucleotide (NMN). Ac‐p53, p53, and p21 protein expression levels, cell numbers, and β‐gal staining positivity (Fig. S5A–C, Supporting information) all indicated the partial prevention of Cav‐1 knockdown‐induced cellular senescence. We also observed similar results with pyruvate administration, which restores NAD^+^/NADH ratio (Fig. S5D–F, Supporting information). From these results, we concluded that Cav‐1 knockdown induces cellular senescence via SIRT1 inactivation.

### Cav‐1 knockout accelerates premature senescence in MEFs

**Figure 4:**
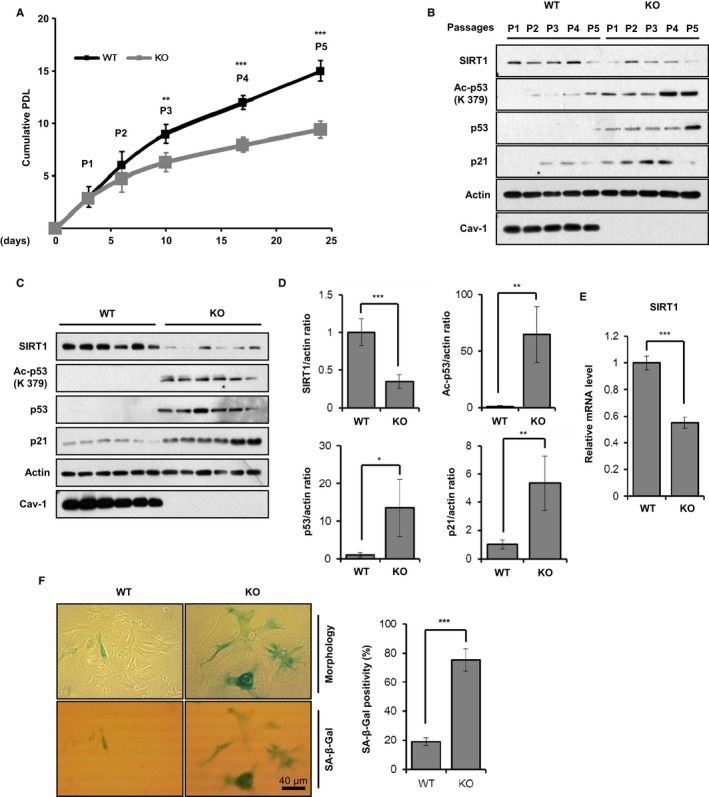
Cav‐1 knockout accelerates premature senescence in MEFs. MEFs were isolated from 14.5‐day embryos of Cav‐1^+/+^ and Cav‐1^*−*/*−*^ mice (*n* = 6 for each group). The cells (3 × 10^4^) were seeded in 6‐well plates and grown for 3–4 days. The cells were counted at each passage. *P* indicates passage number (A). Cav‐1^+/+^ and Cav‐1^*−*/*−*^ MEFs were harvested at the indicated passage number and were subjected to immunoblotting analysis (B). Cav‐1^+/+^ and Cav‐1^*−*/*−*^ MEFs at passage number 3 were harvested (C–E). Whole cell lysates were analyzed by immunoblotting (C). The statistical significance of the protein expression levels of SIRT1, Ac‐p53, p53, and p21 were determined based on the data obtained in C (*n* = 6) (D). The SIRT1 mRNA expression level was determined by qPCR (*n* = 3) (E). Cellular morphology and β‐Gal staining positivity were determined from Cav‐1^+/+^ and Cav‐1^*−*/*−*^ MEFs at passage number 5 (*n* = 3) (F). All data are shown as the mean ± SD. Statistical significance was determined using Student's *t*‐test. *P <* *0.05, **P <* *0.01, and ***P <* *0.001.

To confirm the Cav‐1 knockdown‐induced cellular senescence, we observed cellular senescence after serial cultivation of MEFs obtained from Cav‐1^+/+^ and Cav‐1^*−*/*−*^ mice. Cav‐1^*−*/*−*^ MEFs showed slow growth rate compared with Cav‐1^+/+^ MEFs during serial cultivation (Fig. 4A). During serial cultivation and at passage number 3, compared with Cav‐1^+/+^ MEFs, SIRT1 protein and mRNA expression levels were significantly decreased, while Ac‐p53, p53, and p21 protein expression levels were increased in Cav‐1^*−*/*−*^ MEFs (Fig. 4B–E). In addition, at passage number 5, Cav‐1^*−*/*−*^ MEFs had more β‐gal‐positive cells than Cav‐1^+/+^ MEFs (Fig. 4F). Thus, we concluded that premature senescence is accelerated by Cav‐1 knockout.

**Figure 5:**
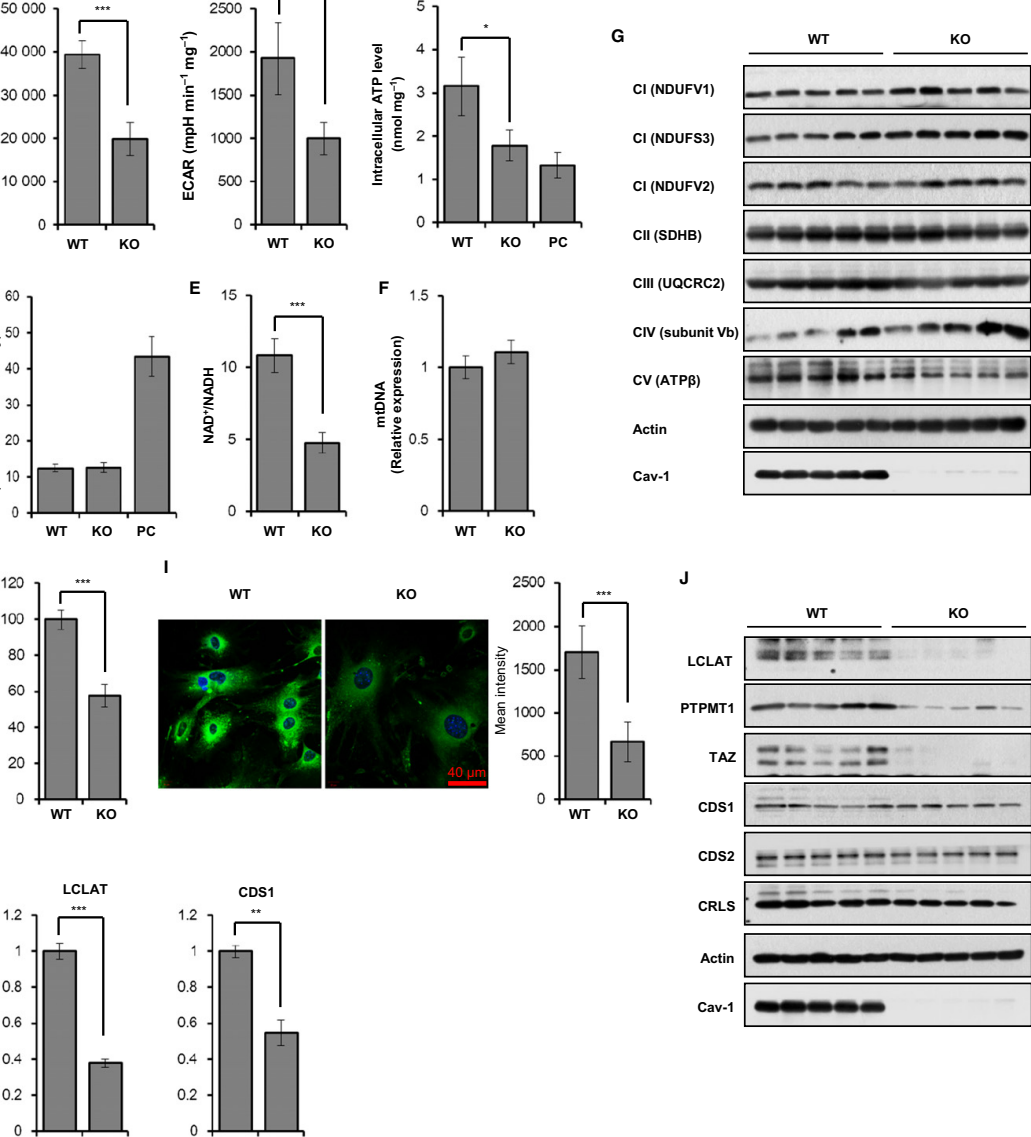
Cav‐1 knockout induces mitochondrial dysfunction in MEFs. MEFs were isolated from 14.5‐day embryos of Cav‐1^+/+^ and Cav‐1^*−*/*−*^ mice and grown at passage number 5 (A–I). The OCR (A) and ECAR (B) were measured using an XF24 analyzer and normalized to protein concentration. The intracellular ATP level was measured using a luminescent luciferase assay, and the ATP content was normalized to the protein amount. For a positive control, the cells were treated with 5 μg mL^−1^ oligomycin for 6 h (C). Intracellular ROS generation was analyzed by FACS after staining cells with 5 μm MitoSox red for 15 min. For a positive control, the cells were treated with 0.5 μg mL^−1^ doxorubicin for 12 h (D). Intracellular NAD ^+^ and NADH levels were measured using a quantitation colorimetric assay kit (E). Genomic DNA (RPS 18) and mitochondrial DNA (mtDNA, COX2) content were determined by qPCR. COX2 mtDNA content was normalized to RPS 18 genomic DNA (F). Immunoblotting was performed for CI (NDUFV1 and V2 and NDUFS3), CII (SDHB), CIII (UQCRC2), CIV (subunit Vb), and CV (ATPβ) using actin as a loading control (G). CI activity was assessed for Cav‐1^+/+^ and Cav‐1^*−*/*−*^ MEFs at passage number 5 (H). The cells were stained with 100 nm NAO for 30 min. Fluorescence intensity was measured using ZEN2009 software (I). LCLAT and CDS1 mRNA expression levels and LCLAT, CDS1, CDS2, CRLS, PTPMT1, TAZ, and Cav‐1 protein expression levels were determined in Cav‐1^+/+^ and Cav‐1^*−*/*−*^ MEFs at passage number 3 by qPCR (J) and immunoblotting (K), respectively. All data are shown as the mean ± SD. Statistical significance was determined using Student's *t*‐test. *P <* *0.05, **P <* *0.01, and ***P <* *0.001.

The OCR, ECAR, intracellular ATP level, and NAD^+^/NADH ratio were significantly reduced in Cav‐1^*−*/*−*^ MEFs compared with Cav‐1^+/+^ MEFs (Fig. 5A–E). However, the ROS level was not different between Cav‐1^+/+^ and Cav‐1^*−*/*−*^ MEFs. Although the mtDNA content and the expression levels of various subunits of CI‐CV were similar in both Cav‐1^+/+^ and Cav‐1^*−*/*−*^ MEFs (Fig. 5F,G), the enzymatic activity of CI was reduced to approximately 57% in Cav‐1^*−*/*−*^ MEFs compared with Cav‐1^+/+^ MEFs (Fig. 5H). In addition, the level of cardiolipin, which was measured by NAO fluorescence intensity, was significantly decreased in Cav‐1^*−*/*−*^ MEFs compared with Cav‐1^+/+^ MEFs (Fig. 5I).

To elucidate how Cav‐1 knockout decreases cardiolipin content, we measured the protein and mRNA expression levels of cardiolipin biosynthesis enzymes. As shown in Fig. 5J, the lysocardiolipin acyltransferase 1 (LCLAT) protein expression level, the amount of protein tyrosine phosphatase localized to the mitochondrion 1 (PTPMT1), and tafazzin (TAZ) were significantly decreased in Cav‐1^*−*/*−*^ MEFs compared with Cav‐1^+/+^ MEFs. The decreased protein and mRNA level in cardiplipin biosynthesis enzymes was similar in Cav‐1‐knockdowned A549 cells (Fig. S6B,C, Supporting information). The LCLAT and CDP‐diacylglycerol synthase 1 (CDS1) mRNA expression levels were also reduced in Cav‐1^*−*/*−*^ MEFs (Fig. 5K). mRNA level of other enzymes was not significantly changed (Fig. S6A, Supporting information). Based on these results, we concluded that Cav‐1 is necessary for the regulation of cardiolipin biosynthesis.

### Cav‐1 knockdown prevents tumor growth in a xenograft mouse model

**Figure 6:**
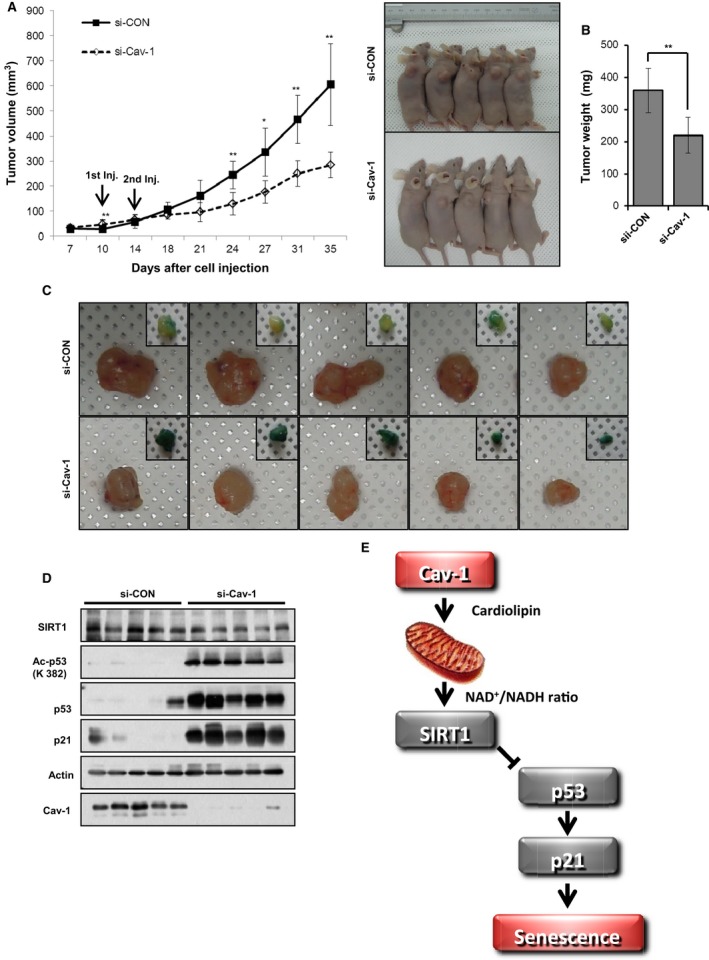
Cav‐1 knockdown prevents tumor growth in a xenograft mouse model. A549 cells (5 × 10^6^) were subcutaneously injected into BALB/c athymic mice (A–D). Tumor size was measured for 35 days after the injection of either si‐CON or si‐Cav‐1 on the 10^th^ and 14^th^ days. Tumor volume in the xenograft mice (*n* = 5) was measured at the indicated times. The right panel shows photographs of the tumor‐bearing mice (A). Tumor weight was determined after tumor isolation (B). The isolated tumors were photographed, and partial slices were stained with X‐gal (C). The isolated tumors were assessed by immunoblotting for SIRT1, Ac‐p53, p53, p21, and Cav‐1 expression using actin as a loading control (D). A schematic model describing how Cav‐1 deficiency induces cellular senescence. Cav‐1 is necessary for activating mitochondrial oxidative phosphorylation by positively regulating cardiolipin biosynthesis. Thus, Cav‐1 activates SIRT1 with a high NAD ^+^/NADH ratio, preventing senescence (E). All data are shown as the mean ± SD. Statistical significance was determined using Student's *t*‐test. *P <* *0.05 and **P <* *0.01.

To confirm the biological effects of Cav‐1 deficiency‐induced premature senescence *in vivo*, we examined the effects of Cav‐1 knockdown in a xenograft tumor mouse model. A549 cells were subcutaneously injected into BALB/c athymic mice. Tumor size was measured for 35 days after the injection of si‐control or si‐Cav‐1 on the 10^th^ and 14^th^ days. As determined by tumor volume and weight, tumor growth was retarded by Cav‐1 knockdown (Fig. 6A,B). The β‐gal staining and Ac‐p53, p53, p21, and Cav‐1 protein expression levels in each tumor showed that Cav‐1 knockdown retarded tumor growth by inducing the cellular senescence of A549 cells (Fig. 6C,D). Similar results were obtained using H460 cells (Fig. S7, Supporting information).

## Discussion

Because there are conflicting observations regarding the role of Cav‐1 in premature senescence, we re‐examined whether Cav‐1 was necessary for cellular senescence after Cav‐1 knockdown or knockout. Our results show that Cav‐1 deficiency leads to cellular senescence via mitochondrial dysfunction. As summarized in Fig. 6E, Cav‐1 is required for expressing cardiolipin biosynthesis enzymes, such as LCLAT and PTPMT1. Cardiolipin is indispensable for oxidative phosphorylation in mitochondria. In the presence of Cav‐1, the active oxidative phosphorylation in mitochondria increases the NAD^+^/NADH ratio and activates SIRT1, which consequently deacetylates, destabilizes, and degrades p53, inactivating a p53‐p21 pathway and preventing premature senescence.

Contradicting observations by other groups have demonstrated that both reduction and increase in Cav‐1 is linked to premature senescence. For example, Cav‐1 is required for oxidative stress‐induced premature senescence (Volonte *et al*., 2002; Bartholomew *et al*., 2009). On the contrary, Cav‐1 knockdown induces a senescence‐like morphological change and prevents *in vitro* and *in vivo* cellular growth in human colorectal cancer cells (Chretien *et al*., 2008; Ha *et al*., 2012; Madaro *et al*., 2013). It is possible that Cav‐1 may contribute differently upon different stress or senescence inducers in different cell types, because our study did not involve transient stresses such as ROS by hydrogen peroxide or DNA damage by bleomycin to induce senescence. Moreover, our observations were not limited to A549 cells. Cav‐1 knockdown‐induced senescence was explored in HCT116 human colorectal carcinoma cells, human diploid fibroblasts (HDFs), and H460 human lung cancer cells with similar results (Fig. S2, Supporting information). In addition, increased Cav‐1 expression level in old animals and elderly people (Park *et al*., 2000; Kang *et al*., 2006) could be simply the effect of aging, not the cause of aging. In contrast to these observations, Cav‐1 deficiency exhibit a premature aging and a pathological phenotypes in tissues (Cohen *et al*., 2003; Head *et al*., 2010; Briand *et al*., 2011; Trajkovski *et al*., 2011). In MEFs and mouse liver, Cav‐1 knockout also leads to mitochondrial dysfunction, which is a strong inducer of premature senescence (Bosch *et al*., 2011; Asterholm *et al*., 2012).

Interestingly, these contradicting results regarding the role of Cav‐1 in senescence is not unique to Cav‐1. For example, both the reduction (Maehara *et al*., 2005; Park *et al*., 2006) and overexpression (Dimri *et al*., 2000) of E2F transcription factor lead to senescence. Furthermore, many regulators of the DNA damage response are implicated in senescence (Bartkova *et al*., 2006; Mallette *et al*., 2007), while their inactivation also leads to senescence (Ju *et al*., 2006).

Cardiolipin content and CI and CIV activity levels decline with age in rat heart and liver and human epidermis, suggesting that the decline of cardiolipin content might be a major culprit for mitochondrial dysfunction in aging (Chicco & Sparagna, 2007). Indeed, cardiolipin administration restores the activity of CI and CIV in dysfunctional mitochondria from aged rat brains and hearts (Chicco & Sparagna, 2007). Our data also demonstrated that the decreased cardiolipin content and CI activity resulted from Cav‐1 knockdown or knockout (Figs 3C and 5H,I) led to mitochondrial dysfunction with reductions in mitochondrial respiration, ATP content, and the NAD^+^/NADH ratio (Fig. 3A–C, Fig. S2, Supporting information and Fig. 6). Because the protein expression level of key enzymes for cardiolipin biosynthesis such as LCLAT, PTPMT1, and TAZ was dramatically decreased in Cav‐1 knockout MEFs (Fig. 5J,K), it is important to understand how Cav‐1 regulates the expression of these enzymes in the future study.

Mitochondrial dysfunction induces cellular senescence by increasing the ROS level, because ROS scavengers prevent stressor‐induced cellular senescence (Wang & Hekimi, 2015). However, our results show that Cav‐1 or NDUFV1 knockdown induced cellular senescence without generating ROS (Figs S2D and S3G, Supporting information). Notably, in C2C12 myotubes, NDUFV1 knockdown does not generate ROS because NDUFV1 is the first subunit to accept electrons from NADH (Hong *et al*., 2014). Based on our results, we concluded that Cav‐1 deficiency induces cellular senescence via an ROS‐independent pathway.

Unexpectedly, we observed Cav‐1 deficiency results in a decreased ECAR (Fig. S2B, Supporting information and Fig. 5B). Senescent HDFs have a reduced conversion of glucose to lactate, compared to young, suggesting that most of the input glucose is used for other purposes in senescent cells (Zwerschke *et al*., 2003). In addition, the upregulated and active p53 in senescence cells antagonizes the uptake of glucose by lowering expression of GLUT1 and induces TIGAR (TP53‐inducible glycolysis and apoptosis regulator), which antagonize the early stage of glycolysis (Wiley & Campisi, 2016). Thus, Cav‐1 knockdown‐induced p53 activation could reduce ECAR by decreasing the expression of GLUT1 but increasing the expression of TIGAR.

Mitochondria differently regulate cellular senescence, depending on cellular metabolic profiles. For example, BRAF‐induced senescent cells exhibit increased TCA cycle activity with pyruvate consumption compared to control cells (Kaplon *et al*., 2013). With higher TCA and oxidative phosphorylation activities, the oncogene‐induced senescent cells generate more ROS, which induces DNA damage response (Sun *et al*., 2016). In the case of mitochondrial dysfunction‐associated senescence (MiDAS), the decreased NAD^+^/NADH ratio works as a key factor for senescence induction by SIRT1 inactivation and p53 (Wiley & Campisi, 2016). As discussed in Ziegler *et al*. (2015), it is still controversial which change in glucose metabolism is a consequence of cellular senescence. It is also possible that this change in glucose metabolism is a cause of cellular senescence.

The NAD^+^/NADH ratio and SIRT1‐mediated p53 deacetylation were dramatically decreased after knocking down Cav‐1 (Fig. 3A,D). The SIRT1 mRNA and protein expression levels were also significantly decreased in Cav‐1^*−*/*−*^ MEFs compared with Cav‐1^+/+^ MEFs (Fig. 4B, C and E). Because the administration of SIRT1 activators such as SRT1720 and NMN abolished Cav‐1 knockdown‐induced cellular senescence (Fig. 3E–G and Fig. S4, Supporting information), Cav‐1 is necessary for SIRT1 activation and for protecting cells from senescence. SIRT1 functions as a positive regulator of insulin signaling because it enhances the insulin‐elicited tyrosine phosphorylation of IRβ and IRS‐1 by repressing the transcription of PTP1B, a phosphatase that prevents the tyrosine phosphorylation and activation of IRβ and IRS‐1 (Sun *et al*., 2007; Hong *et al*., 2014). Cav‐1 knockout and Cav‐1‐targeted microRNA contribute to the development of insulin resistance by blocking insulin‐elicited tyrosine phosphorylation and activation of IRβ (Trajkovski *et al*., 2011). However, the molecular mechanism by which Cav‐1 enhances insulin‐elicited tyrosine phosphorylation of IRβ remains elusive. Based on our results, we propose that Cav‐1 enhances insulin‐elicited tyrosine phosphorylation of IRβ via SIRT1 activation and PTP1B repression.

## Experimental procedure

### β‐Gal staining

Cells and isolated tumors were washed twice with PBS and fixed with 3.7% formaldehyde for 5 min. Fixed cells were incubated overnight in the following staining solution: 150 mm NaCl, 2 mm MgCl_2_, 1 mg mL^−1^ X‐Gal, 100 mm citric acid, 100 mm sodium phosphate, pH 6.0, 5 mm potassium ferrocyanide, and 5 mm potassium ferricyanide.

### Measurement of intracellular ATP content

Intracellular ATP was measured as described by Hong *et al*. (2014). Cell lysates using NaOH and HCl were subjected to an ATP assay according to the manufacturer's protocol (Sigma‐Aldrich, St Louis, MO, USA).

### Measurement ROS

Cells were incubated in cultured media MitoSox red (5 μm, Invitrogen, Camarillo, CA, USA) for 15 min, 37 °C, collected by trypsinization, and washed with PBS. Stained cells were analyzed using a FACS Calibur flow cytometer (BD Bioscience, San Diego, CA, USA).

### Determination of NAD^+^ and NADH concentrations

NAD^+^ and NADH concentrations and the NAD^+^/NADH ratio were determined using an NAD^+^/NADH Quantification Colorimetric Kit (Bio Vision, Milpitas, CA, USA). Total NADH and NAD^+^ (NADt) and NADH were measured in the assay. The amount of NAD^+^ was obtained by subtracting the amount of NADH from the NADt.

### Assay for oxygen consumption rate and extracellular acidification rate

The mitochondrial OCR and ECAR were analyzed using an XF24 Extracellular Flux Analyzer (Seahorse Bioscience, Santa Clara, CA, USA) as described previously (Lee *et al*., 2016).

### Assay for mitochondrial respiratory chain activities

Mitochondrial respiratory chain activities were measured as described previously (Spinazzi *et al*., 2012). Complex I activity was measured using an enriched mitochondrial fraction; other complexes were measured using cultured cells. Detached cells were washed with PBS and centrifuged at 1000* *× *g* for 5 min at 4 °C. The cell pellet was homogenized in 10 mm ice‐cold hypotonic Tris buffer, pH 7.6, and then mixed with a 1.5 m sucrose solution. The mixed solution was centrifuged at 600* *× *g* for 10 min at 2 °C; the supernatant was transferred to a new tube and centrifuged at 14 000* *× *g* for 10 min at 2 °C. The mitochondrial pellet was resuspended with 10 mm ice‐cold hypotonic Tris buffer, pH 7.6, and then subjected to the assay.

Supplementary information contains other experimental procedures (Appendix S1), supplementary figures, figure legends, and tables (Table S1‐S3).

## Funding

This work was supported by grants awarded to Y.‐G. Ko from the National Research Foundation (2011‐0017562 and 2015R1A5A1009024).

## Conflict of interest

None declared.

## Author contributions

D.‐M.Y. performed the majority of the *in vitro* experiments. S.H.J. and H.‐T.A. performed the cell cycle analysis and animal experiments, respectively. J.H. and J.S.P. performed the OCR and ECAR measurements and the qPCR analysis, respectively. S.L. designed and supervised radioactive labeling experiment and revised the manuscript. H.L. performed the cardiolipin analysis. H.L and M.‐S.B. isolated MEFs from Cav‐1 knockout mice. H.C.L. and N.‐K.H. initiated the experiment for Cav‐1 knockdown‐induced senescence. J.K. supervised the animal experiments. J.‐S.L. provided critical suggestions. Y.‐G.K. designed and supervised the study and wrote the manuscript.

## Supporting information


**Fig. S1** Caveolin‐1 knockdown‐induced senescence is the si‐Cav‐1 specific effect.
**Fig. S2** Caveolin‐1 knockdown induces premature senescence in various cell lines.
**Fig. S3** Caveolin‐1 knockdown leads to mitochondrial dysfunction.
**Fig. S4** CI dysfunction induces premature senescence.
**Fig. S5** Cav‐1 knockdown‐induced senescence results from SIRT1 inactivation.
**Fig. S6** The mRNA and protein expression levels of enzymes in cardiolipin biosynthesis pathway.
**Fig. S7** Cav‐1 knockdown prevents tumor growth in a xenograft mouse model.

**Table S1** siRNA sequence for each gene.
**Table S2** Primer sequence for each gene.
**Table S3** List of antibodies for immunoblotting (IB).

**Appendix S1** Experimental procedure.
