# Supplementary material for: Caveolin‐1 deficiency induces premature senescence with mitochondrial dysfunction
Source: Aging Cell. 2017 May 17;16(4):773–84. doi: 10.1111/acel.12606 (PMC5506423; doi:10.1111/acel.12606)
Supplement: Supplementary file 1 — Fig. S1 Caveolin‐1 knockdown‐induced senescence is the si‐Cav‐1 specific effect. Fig. S2 Caveolin‐1 knockdown induces premature senescence in various cell lines. Fig. S3 Caveolin‐1 knockdown leads to mitochondrial dysfunction. Fig. S4 CI dysfunction induces premature senescence. Fig. S5 Cav‐1 knockdown‐induced senescence results from SIRT1 inactivation. Fig. S6 The mRNA and protein expression levels of enzymes in cardiolipin biosynthesis pathway. Fig. S7 Cav‐1 knockdown prevents tumor growth in a xenograft mouse model. [file ACEL-16-773-s001.pptx]

## Slide 1
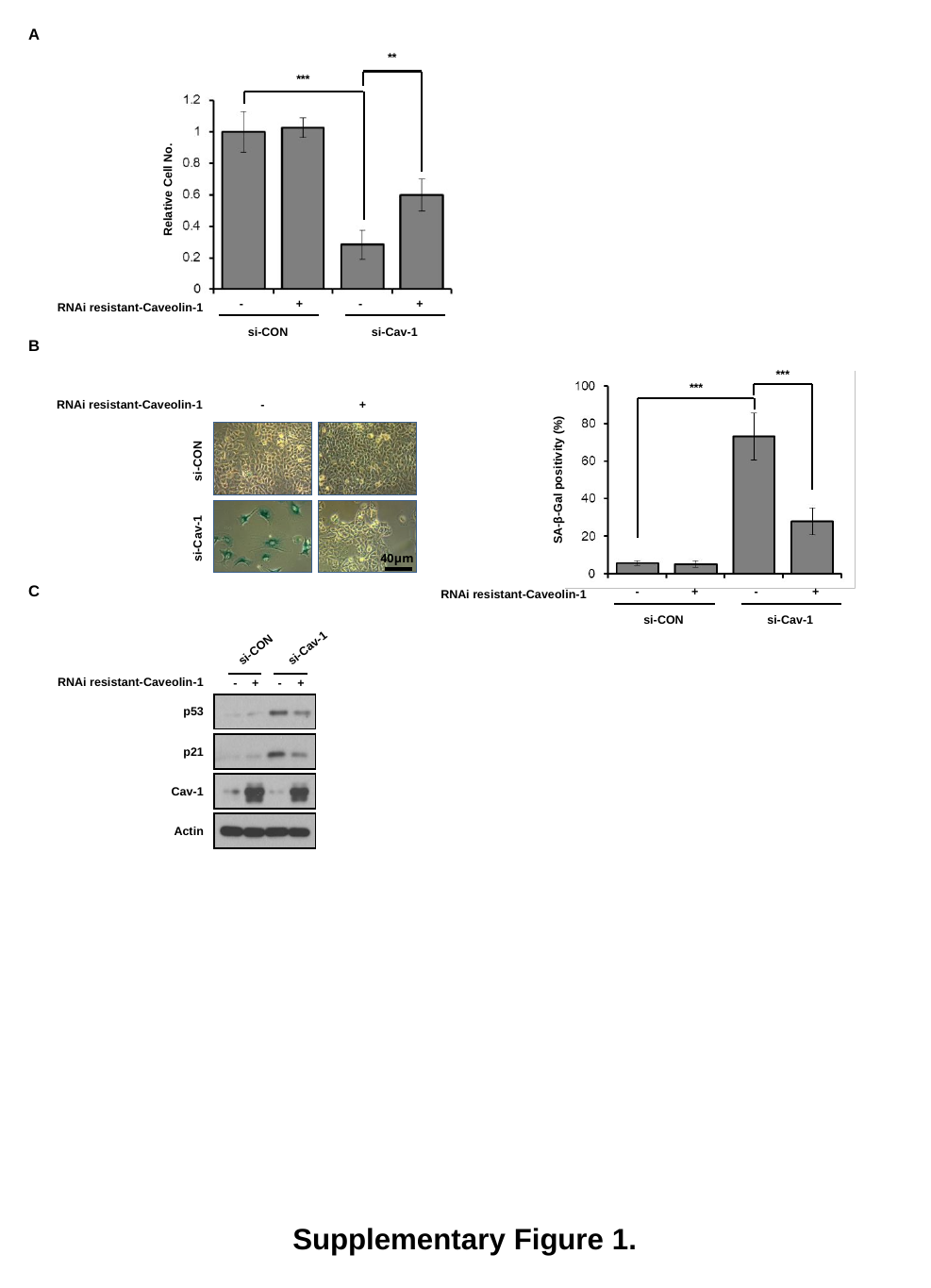

A
**
***
Relative Cell No.
-
+
-
+
RNAi resistant-Caveolin-1
si-CON
si-Cav-1
B
***
***
SA-β-Gal positivity (%)
-
+
-
+
RNAi resistant-Caveolin-1
si-CON
si-Cav-1
RNAi resistant-Caveolin-1
-
+
si-CON
si-Cav-1
40μm
C
si-Cav-1
si-CON
RNAi resistant-Caveolin-1
-
+
-
+
p53
p21
Cav-1
Actin
Supplementary Figure 1.

## Slide 2
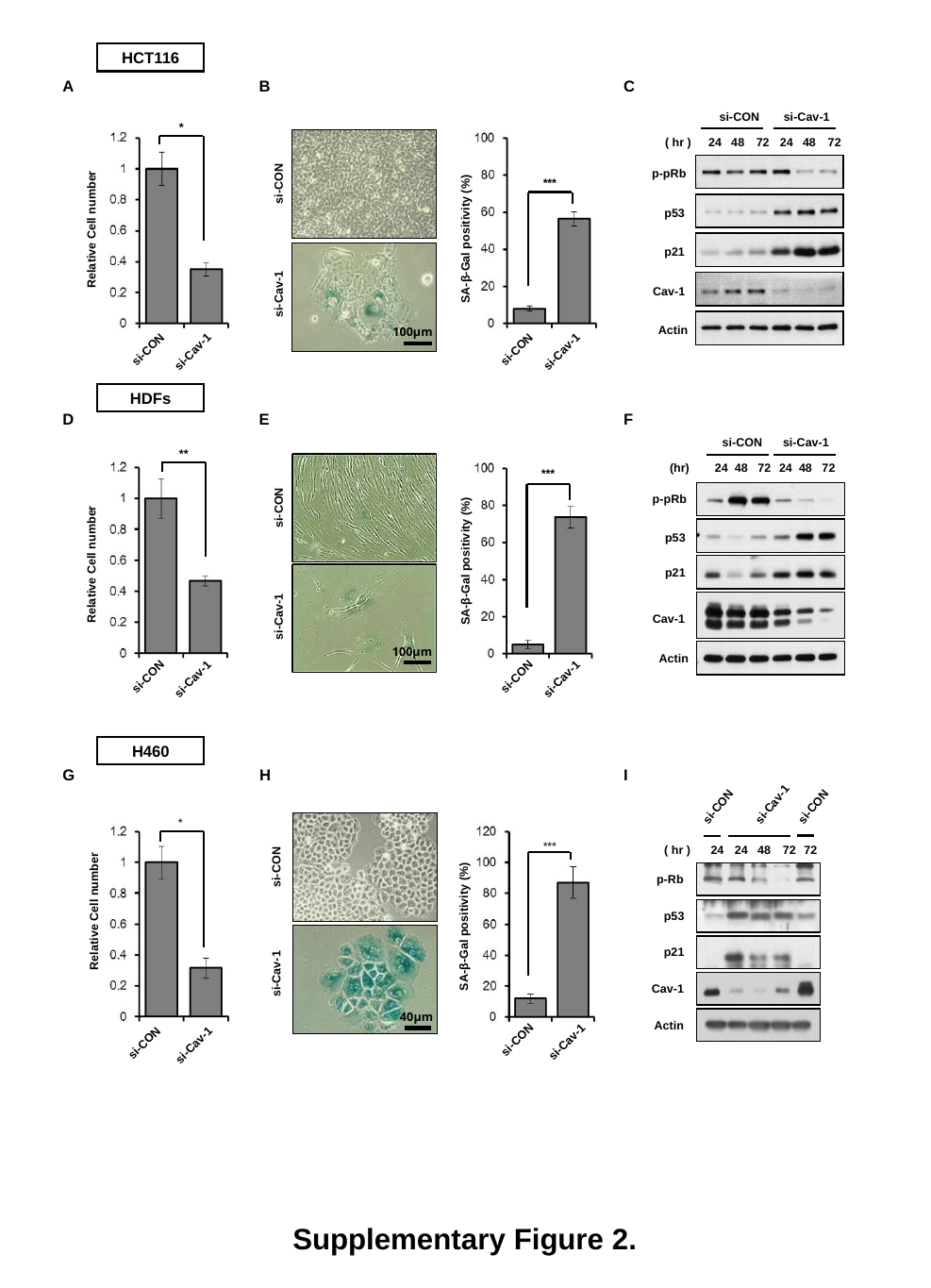

HCT116
C
A
B
si-CON
si-Cav-1
24
48
72
24
48
72
( hr )
p-pRb
p53
p21
Cav-1
Actin
*
***
si-CON
Relative Cell number
SA-β-Gal positivity (%)
si-Cav-1
100μm
si-CON
si-Cav-1
si-CON
si-Cav-1
HDFs
D
E
F
si-Cav-1
si-CON
(hr)
24 48 72
24 48 72
**
***
p-pRb
si-CON
p53
SA-β-Gal positivity (%)
Relative Cell number
p21
Cav-1
si-Cav-1
100μm
Actin
si-CON
si-Cav-1
si-CON
si-Cav-1
H460
G
H
I
si-Cav-1
si-CON
si-CON
72
( hr )
24
24
48
72
*
si-CON
si-Cav-1
***
p-Rb
Relative Cell number
p53
SA-β-Gal positivity (%)
p21
Cav-1
40μm
Actin
si-CON
si-Cav-1
si-CON
si-Cav-1
Supplementary Figure 2.

## Slide 3
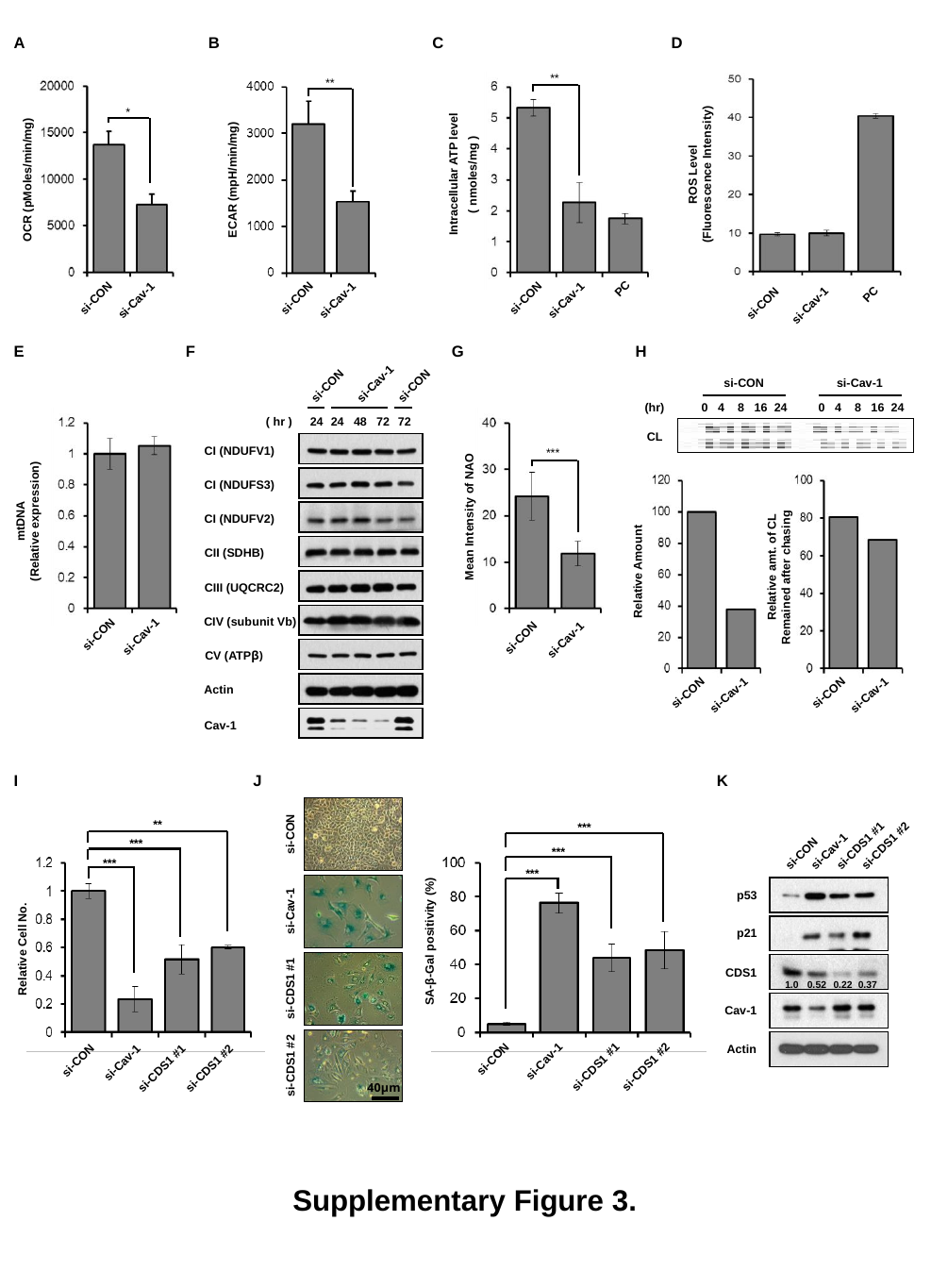

A
B
C
D
**
Intracellular ATP level
( nmoles/mg )
PC
si-CON
si-Cav-1
ROS Level
(Fluorescence Intensity)
PC
si-CON
si-Cav-1
**
ECAR (mpH/min/mg)
si-CON
si-Cav-1
*
OCR (pMoles/min/mg)
si-CON
si-Cav-1
E
F
G
H
si-Cav-1
si-CON
si-CON
72
24
24
48
72
( hr )
CI (NDUFV1)
CI (NDUFS3)
CI (NDUFV2)
CII (SDHB)
CIII (UQCRC2)
CIV (subunit Vb)
CV (ATPβ)
Actin
Cav-1
si-CON
si-Cav-1
(hr)
0 4 8 16 24
0 4 8 16 24
CL
***
Mean Intensity of NAO
si-CON
si-Cav-1
mtDNA
(Relative expression)
Relative Amount
Relative amt. of CL
Remained after chasing
si-CON
si-Cav-1
si-CON
si-CON
si-Cav-1
si-Cav-1
I
J
K
si-CON
si-Cav-1
si-CDS1 #1
si-CDS1 #2
40μm
**
***
***
si-CDS1 #1
si-CDS1 #2
si-Cav-1
***
si-CON
***
***
p53
p21
SA-β-Gal positivity (%)
Relative Cell No.
CDS1
1.0
0.52
0.22
0.37
Cav-1
Actin
si-CON
si-CON
si-Cav-1
si-Cav-1
si-CDS1 #1
si-CDS1 #2
si-CDS1 #1
si-CDS1 #2
Supplementary Figure 3.

## Slide 4
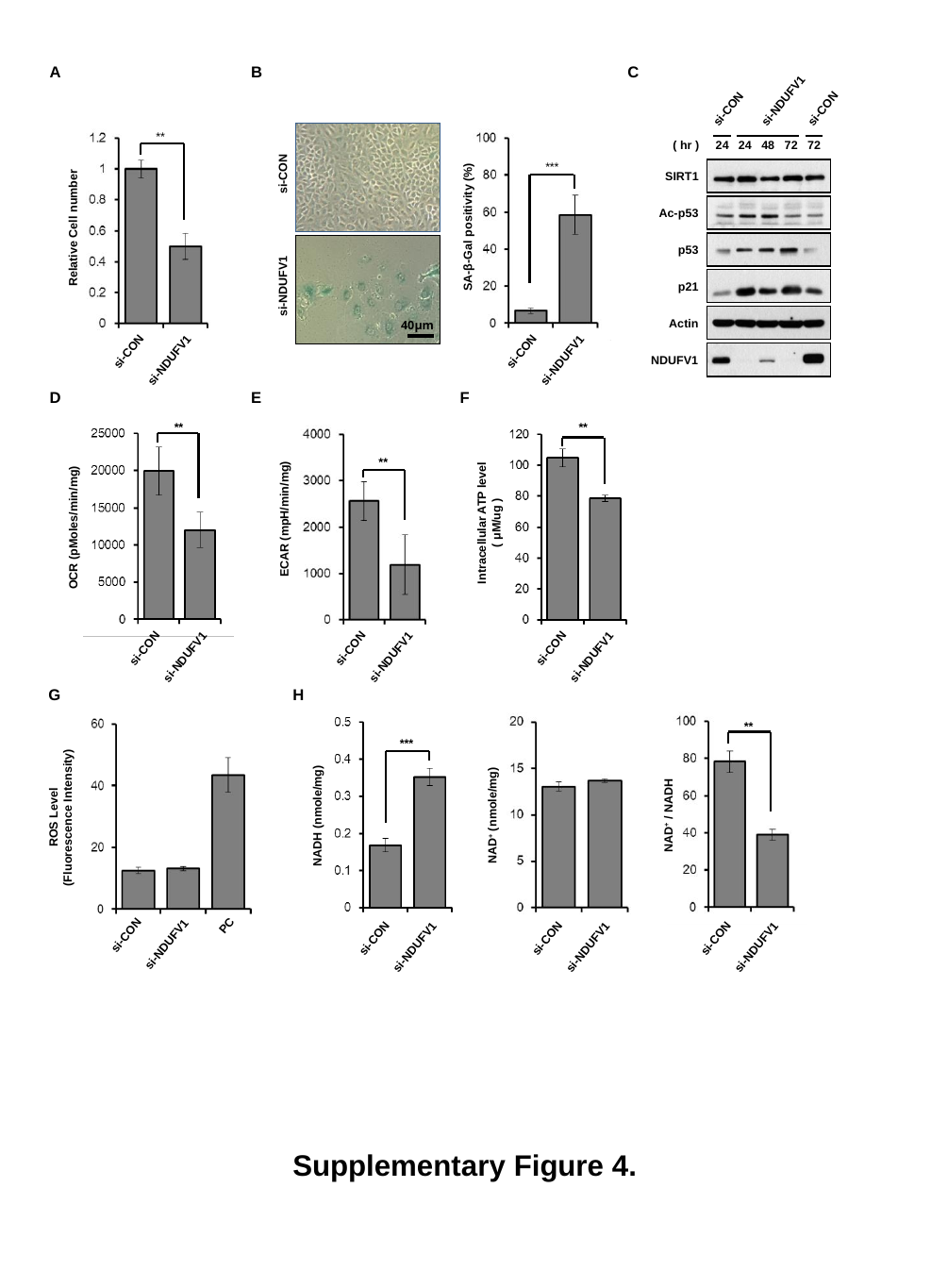

A
B
C
si-CON
si-CON
( hr )
24
24
48
72
72
SIRT1
Ac-p53
p53
p21
Actin
NDUFV1
si-NDUFV1
**
Relative Cell number
si-CON
si-NDUFV1
***
SA-β-Gal positivity (%)
si-CON
si-NDUFV1
si-CON
si-NDUFV1
40μm
D
E
F
**
**
OCR (pMoles/min/mg)
si-CON
si-NDUFV1
**
ECAR (mpH/min/mg)
si-CON
si-NDUFV1
Intracellular ATP level
( μM/ug )
si-CON
si-NDUFV1
G
H
**
NAD+ / NADH
si-CON
si-NDUFV1
***
NADH (nmole/mg)
si-CON
si-NDUFV1
NAD+ (nmole/mg)
si-CON
si-NDUFV1
ROS Level
(Fluorescence Intensity)
PC
si-CON
si-NDUFV1
Supplementary Figure 4.

## Slide 5
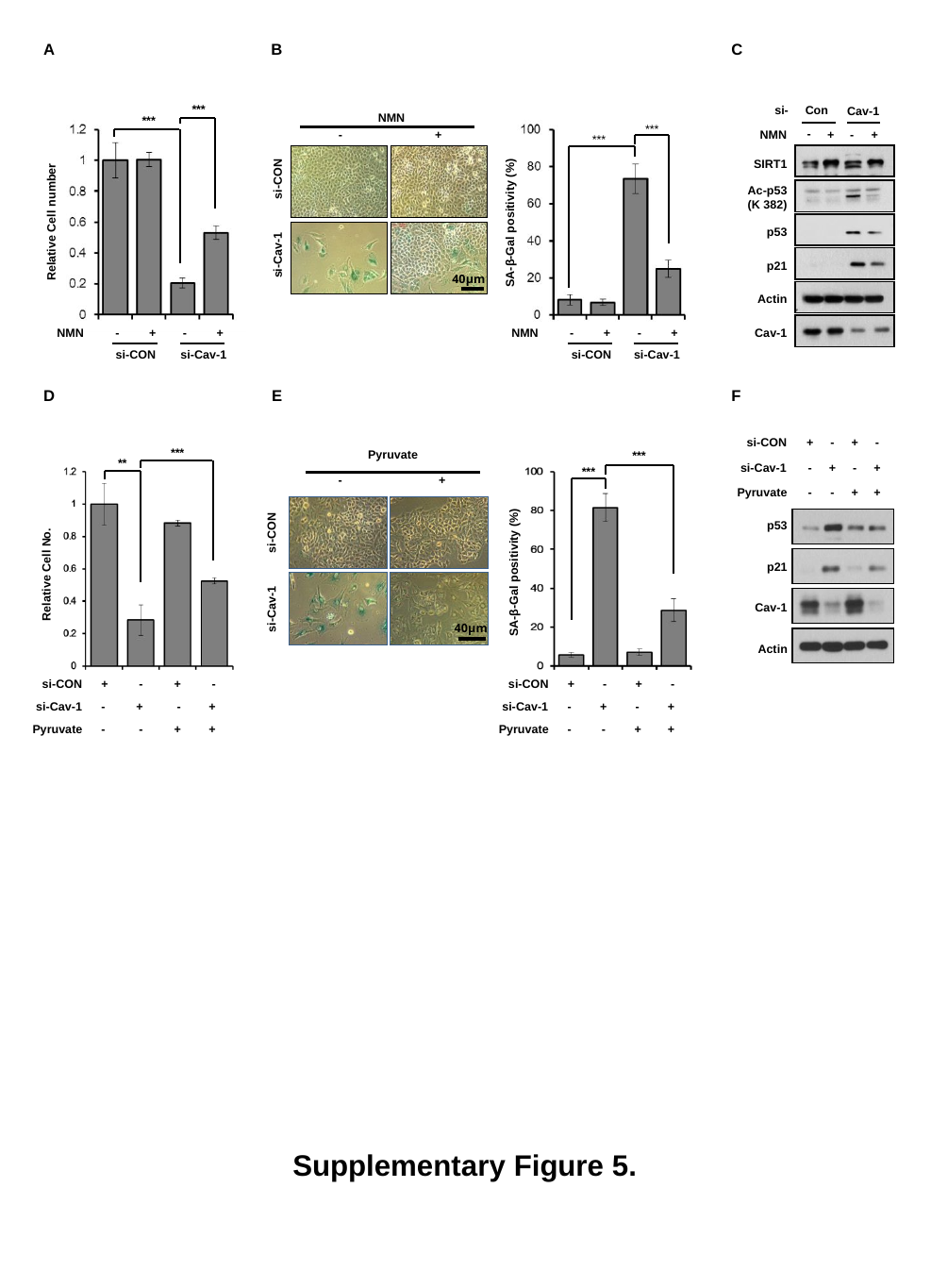

A
B
C
***
si-
Con
Cav-1
NMN
- +
si-CON
si-Cav-1
40μm
***
***
-
+
NMN
-
+
***
SIRT1
Ac-p53
(K 382)
Relative Cell number
SA-β-Gal positivity (%)
p53
p21
Actin
Cav-1
NMN
- +
- +
NMN
- +
- +
si-CON
si-Cav-1
si-CON
si-Cav-1
D
E
F
si-CON
+
-
+
-
***
Pyruvate
-
+
si-CON
si-Cav-1
40μm
***
**
-
+
-
+
si-Cav-1
***
Relative Cell No.
si-CON
+
-
+
-
si-Cav-1
-
+
-
+
Pyruvate
-
-
+
+
Pyruvate
-
-
+
+
p53
p21
SA-β-Gal positivity (%)
Cav-1
Actin
si-CON
+
-
+
-
si-Cav-1
-
+
-
+
Pyruvate
-
-
+
+
Supplementary Figure 5.

## Slide 6
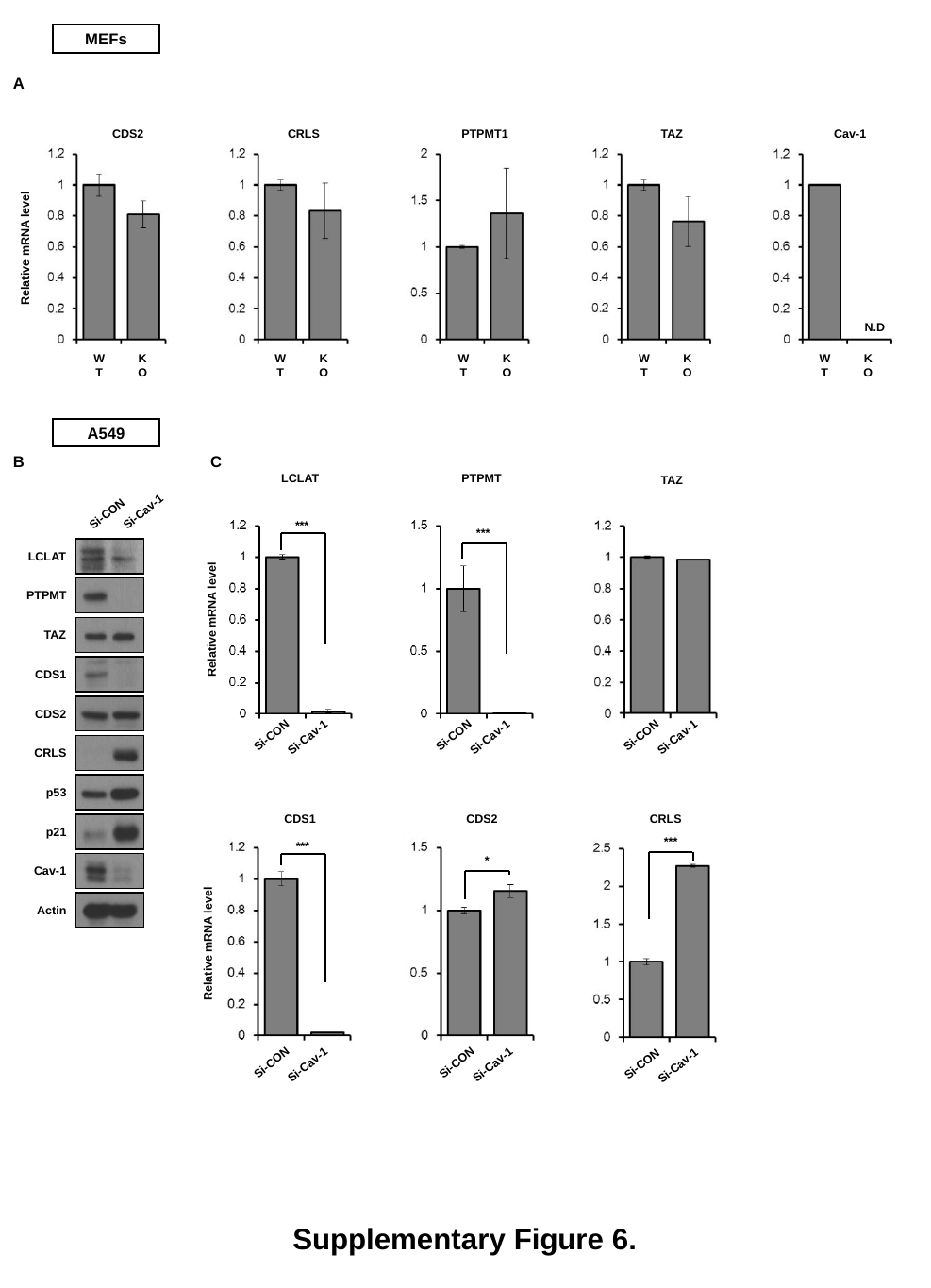

MEFs
A
CDS2
CRLS
PTPMT1
TAZ
Cav-1
Relative mRNA level
N.D
KO
WT
KO
WT
KO
WT
KO
WT
KO
WT
A549
B
C
LCLAT
PTPMT
TAZ
Si-Cav-1
Si-CON
LCLAT
PTPMT
TAZ
CDS1
CDS2
CRLS
p53
p21
Cav-1
Actin
***
Relative mRNA level
Si-CON
Si-Cav-1
***
Si-CON
Si-CON
Si-Cav-1
Si-Cav-1
CDS1
CDS2
CRLS
***
***
*
Relative mRNA level
Si-CON
Si-CON
Si-CON
Si-Cav-1
Si-Cav-1
Si-Cav-1
Supplementary Figure 6.

## Slide 7
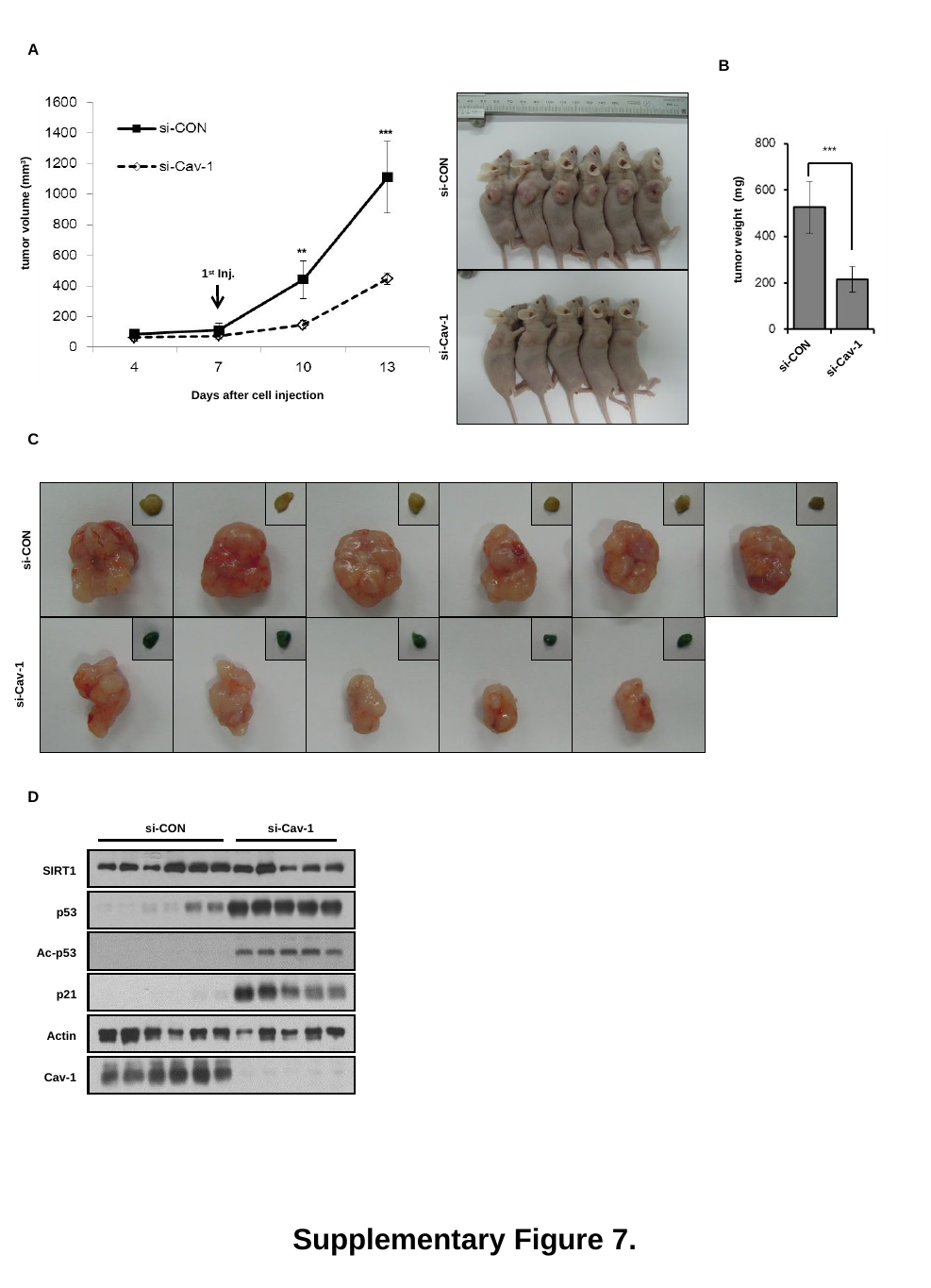

A
B
tumor volume (mm3)
1st Inj.
Days after cell injection
si-CON
si-Cav-1
***
***
tumor weight (mg)
**
si-CON
si-Cav-1
C
si-CON
si-Cav-1
D
si-Cav-1
si-CON
SIRT1
p53
Ac-p53
p21
Actin
Cav-1
Supplementary Figure 7.
