# Supplementary material for: Caveolin‐1 deficiency induces premature senescence with mitochondrial dysfunction
Source: Aging Cell. 2017 May 17;16(4):773–84. doi: 10.1111/acel.12606 (PMC5506423; doi:10.1111/acel.12606)
Supplement: Supplementary file 2 — Table S1 siRNA sequence for each gene. Table S2 Primer sequence for each gene. Table S3 List of antibodies for immunoblotting (IB). [file ACEL-16-773-s002.pptx]

## Slide 1
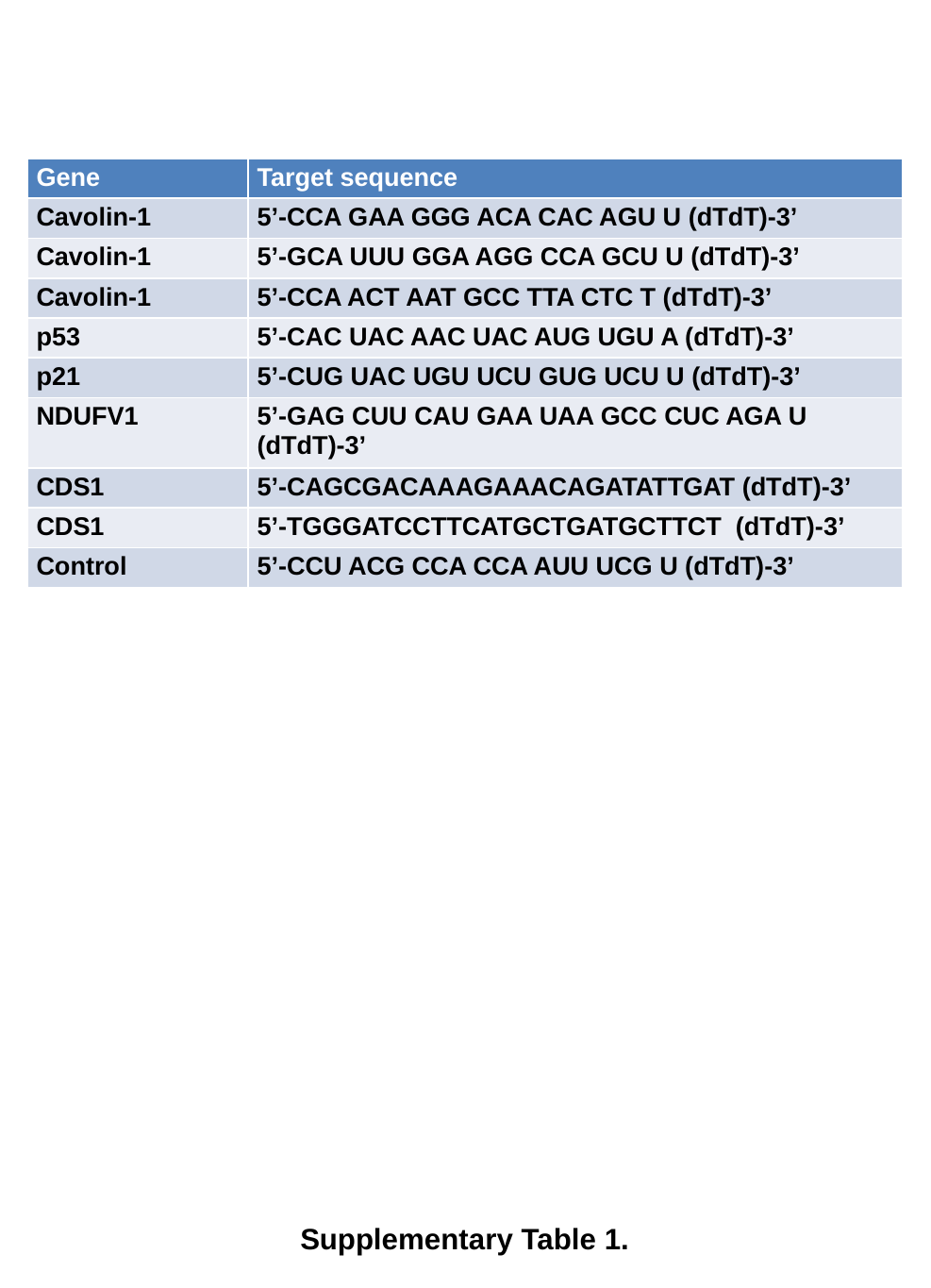

| Gene | Target sequence |
| --- | --- |
| Cavolin-1 | 5’-CCA GAA GGG ACA CAC AGU U (dTdT)-3’ |
| Cavolin-1 | 5’-GCA UUU GGA AGG CCA GCU U (dTdT)-3’ |
| Cavolin-1 | 5’-CCA ACT AAT GCC TTA CTC T (dTdT)-3’ |
| p53 | 5’-CAC UAC AAC UAC AUG UGU A (dTdT)-3’ |
| p21 | 5’-CUG UAC UGU UCU GUG UCU U (dTdT)-3’ |
| NDUFV1 | 5’-GAG CUU CAU GAA UAA GCC CUC AGA U (dTdT)-3’ |
| CDS1 | 5’-CAGCGACAAAGAAACAGATATTGAT (dTdT)-3’ |
| CDS1 | 5’-TGGGATCCTTCATGCTGATGCTTCT (dTdT)-3’ |
| Control | 5’-CCU ACG CCA CCA AUU UCG U (dTdT)-3’ |
Supplementary Table 1.

## Slide 2
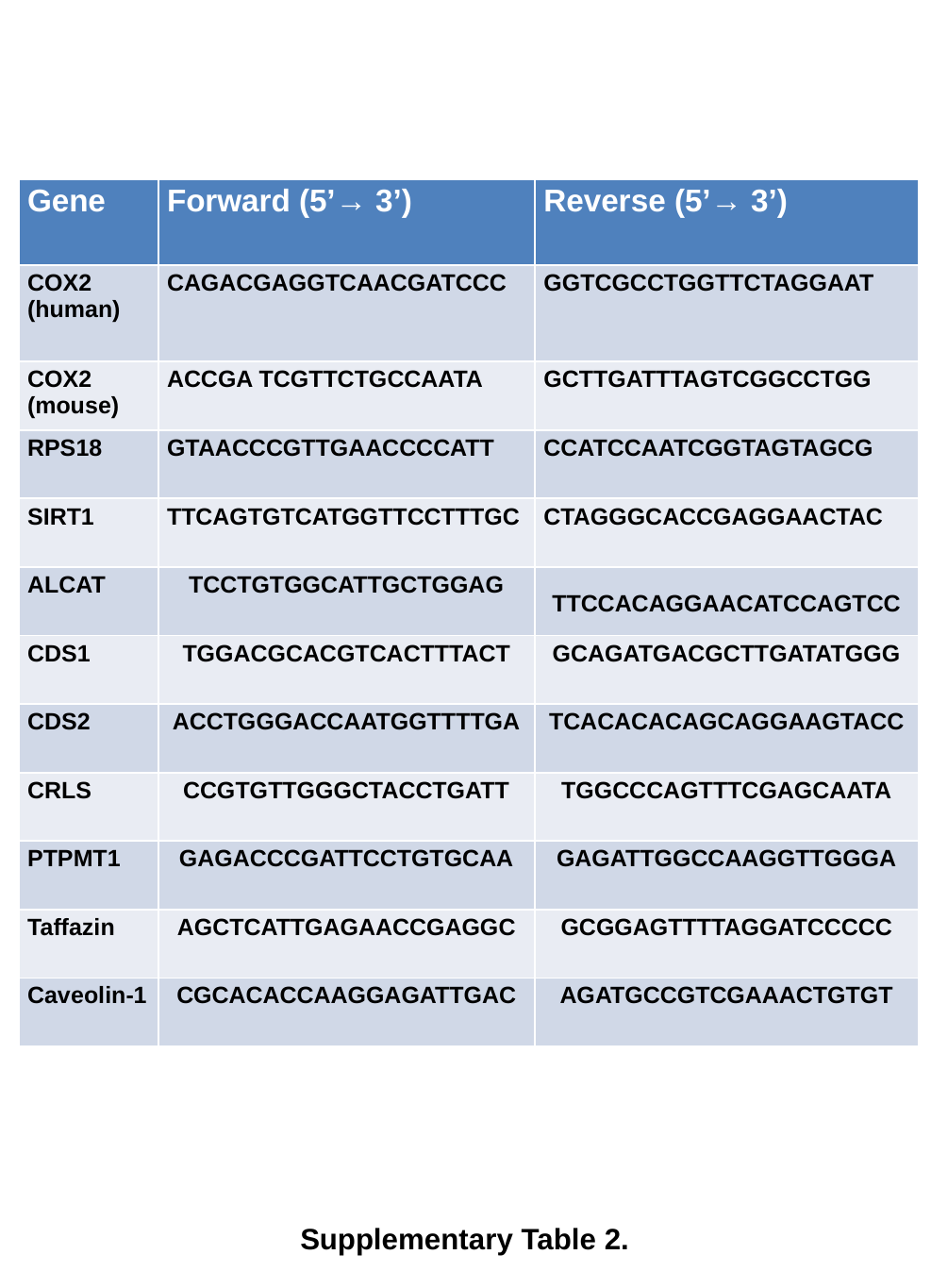

| Gene | Forward (5’→ 3’) | Reverse (5’→ 3’) |
| --- | --- | --- |
| COX2 (human) | CAGACGAGGTCAACGATCCC | GGTCGCCTGGTTCTAGGAAT |
| COX2 (mouse) | ACCGA TCGTTCTGCCAATA | GCTTGATTTAGTCGGCCTGG |
| RPS18 | GTAACCCGTTGAACCCCATT | CCATCCAATCGGTAGTAGCG |
| SIRT1 | TTCAGTGTCATGGTTCCTTTGC | CTAGGGCACCGAGGAACTAC |
| ALCAT | TCCTGTGGCATTGCTGGAG | TTCCACAGGAACATCCAGTCC |
| CDS1 | TGGACGCACGTCACTTTACT | GCAGATGACGCTTGATATGGG |
| CDS2 | ACCTGGGACCAATGGTTTTGA | TCACACACAGCAGGAAGTACC |
| CRLS | CCGTGTTGGGCTACCTGATT | TGGCCCAGTTTCGAGCAATA |
| PTPMT1 | GAGACCCGATTCCTGTGCAA | GAGATTGGCCAAGGTTGGGA |
| Taffazin | AGCTCATTGAGAACCGAGGC | GCGGAGTTTTAGGATCCCCC |
| Caveolin-1 | CGCACACCAAGGAGATTGAC | AGATGCCGTCGAAACTGTGT |
Supplementary Table 2.

## Slide 3
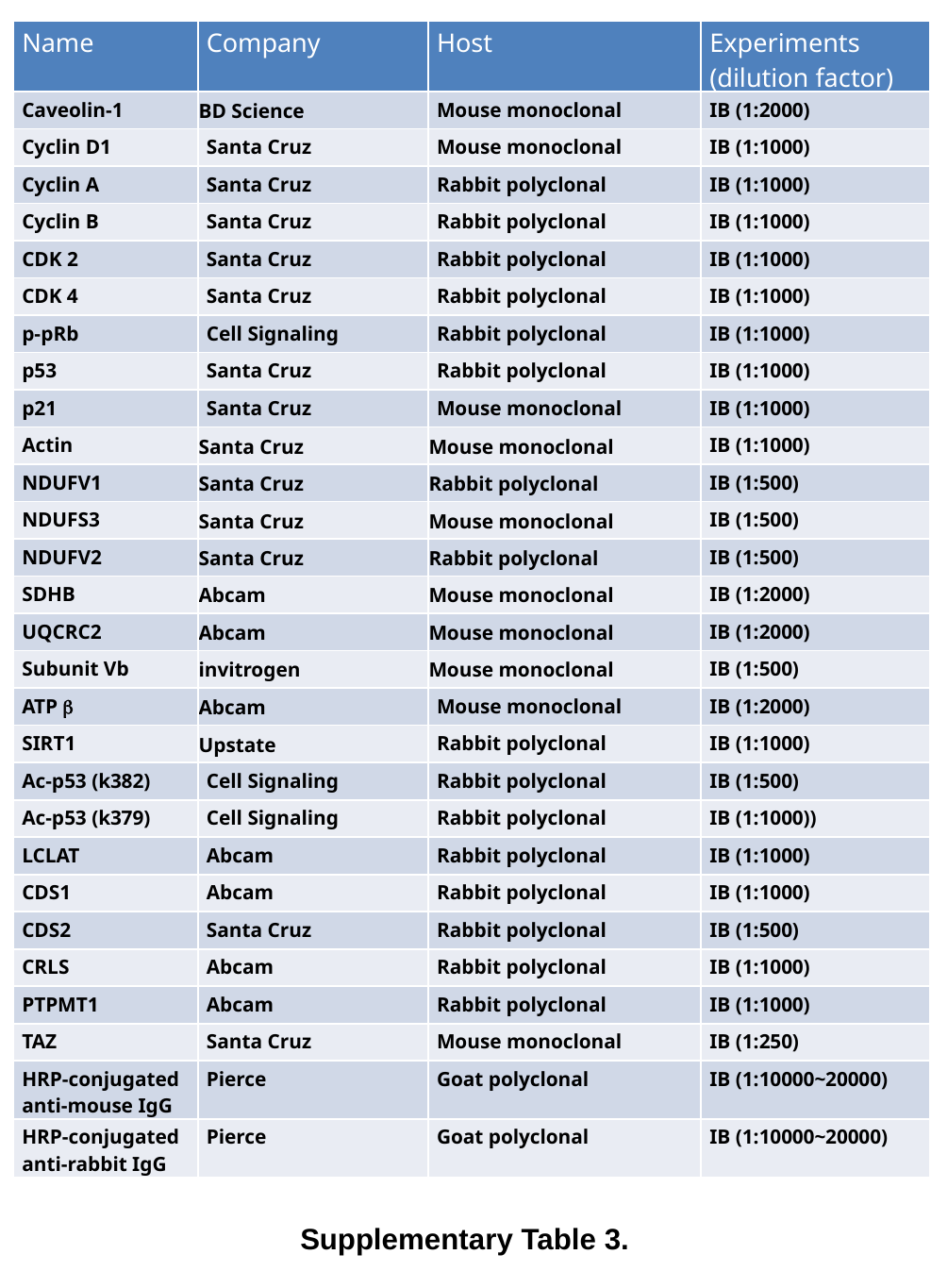

| Name | Company | Host | Experiments (dilution factor) |
| --- | --- | --- | --- |
| Caveolin-1 | BD Science | Mouse monoclonal | IB (1:2000) |
| Cyclin D1 | Santa Cruz | Mouse monoclonal | IB (1:1000) |
| Cyclin A | Santa Cruz | Rabbit polyclonal | IB (1:1000) |
| Cyclin B | Santa Cruz | Rabbit polyclonal | IB (1:1000) |
| CDK 2 | Santa Cruz | Rabbit polyclonal | IB (1:1000) |
| CDK 4 | Santa Cruz | Rabbit polyclonal | IB (1:1000) |
| p-pRb | Cell Signaling | Rabbit polyclonal | IB (1:1000) |
| p53 | Santa Cruz | Rabbit polyclonal | IB (1:1000) |
| p21 | Santa Cruz | Mouse monoclonal | IB (1:1000) |
| Actin | Santa Cruz | Mouse monoclonal | IB (1:1000) |
| NDUFV1 | Santa Cruz | Rabbit polyclonal | IB (1:500) |
| NDUFS3 | Santa Cruz | Mouse monoclonal | IB (1:500) |
| NDUFV2 | Santa Cruz | Rabbit polyclonal | IB (1:500) |
| SDHB | Abcam | Mouse monoclonal | IB (1:2000) |
| UQCRC2 | Abcam | Mouse monoclonal | IB (1:2000) |
| Subunit Vb | invitrogen | Mouse monoclonal | IB (1:500) |
| ATP b | Abcam | Mouse monoclonal | IB (1:2000) |
| SIRT1 | Upstate | Rabbit polyclonal | IB (1:1000) |
| Ac-p53 (k382) | Cell Signaling | Rabbit polyclonal | IB (1:500) |
| Ac-p53 (k379) | Cell Signaling | Rabbit polyclonal | IB (1:1000)) |
| LCLAT | Abcam | Rabbit polyclonal | IB (1:1000) |
| CDS1 | Abcam | Rabbit polyclonal | IB (1:1000) |
| CDS2 | Santa Cruz | Rabbit polyclonal | IB (1:500) |
| CRLS | Abcam | Rabbit polyclonal | IB (1:1000) |
| PTPMT1 | Abcam | Rabbit polyclonal | IB (1:1000) |
| TAZ | Santa Cruz | Mouse monoclonal | IB (1:250) |
| HRP-conjugated anti-mouse IgG | Pierce | Goat polyclonal | IB (1:10000~20000) |
| HRP-conjugated anti-rabbit IgG | Pierce | Goat polyclonal | IB (1:10000~20000) |
Supplementary Table 3.
