# Supplementary material for: Caveolin‐1 deficiency induces premature senescence with mitochondrial dysfunction
Source: Aging Cell. 2017 May 17;16(4):773–84. doi: 10.1111/acel.12606 (PMC5506423; doi:10.1111/acel.12606)
Supplement: Supplementary file 3 — Appendix S1 Experimental procedure. [file ACEL-16-773-s003.docx]

**Supplementary Information**

**Experimental Procedure**

**Cell culture:** All cells were obtained from the ATCC with in the past 3 years. Cell lines were validated by the ATCC using short tandem repeat (STR) profiling. A549, H460 cells were cultured in RPMI1640 medium (Thermo Scientific, Walthman, MA, USA); HCT116 wild-type, p53^-/-^ and p21^-/-^ isogenic cell lines were cultured in McCoy’s 5A medium (Thermo Scientific); and HDFs, MEFs were cultured in Dulbecco’s modified Eagle’s medium (Thermo Scientific). All the cell lines except the MEFs were supplemented with 10% fetal bovine serum (FBS, Thermo Scientific). The MEFs were supplemented with 20% FBS. All cells were supplemented with 1% penicillin/streptomycin (Thermo Scientific) and were incubated in a 5% CO_2_ incubator at 37°C.

**Animal studies:** Cav-1 knockout mice were purchased from The Jackson Laboratory (Stock NO. 007083, Bar Harbor, ME, USA) and C57BL/6 wild type mice were purchased from Orient Bio Inc (Seongnam, Gyeonggi-do, Korea). All MEFs were derived from 14.5 d embryos. For xenografts experiment, A549 and H460 cells (5x10^6^) were injected subcutaneously into 5-week-old BALB/c nude mice (Orient Bio Inc., n=5 for each group). The lengths (L) and widths (W) of the resulting tumors were measured with calipers. Tumor volumes were calculated as (L x W^2^)/2. When the tumors reached an average volume of approximately 50 mm^3^, si-CON and si-Cav-1 were injected into the tumors using AteloGenes Local Use Quick Gelation (Cosmo Bio, Toyo 2-chome, Koto-ku, Tokyo, Japan) following the user’s manual. After the siRNA injections, tumor volumes were measured over 3- or 4-day intervals. All animal studies were conducted with the approval of the Korea University Institutional Animal Care and Use Committee and the Korea Animal Protection Law.

**Antibodies and materials:** Supplementary Table 3 shows the information for the antibodies used for immunoblotting. NMN, oligomycin, doxorubicin and pyruvate were obtained from Sigma-Aldrich (St Louis, MO, USA). 2’-7’-dichlorofluorescein diacetate and 10-N-nonyl acridine orange (NAO) were obtained from Thermo Fisher Scientific. SRT1720 was obtained from Sellekchem (Houston, TX, USA).

**siRNA interference:** All siRNAs (Supplementary Table 1) were synthesized by Bioneer Inc. (Daejeon, Korea). We transfected 100 nM siRNA into the cells using Lipofectamine RNAiMAX reagent (Invitrogen, Karlsruhe, Germany).

**Immunoblotting:** Cells were lysed with the following lysis buffer: 50 mM Tris-Cl, pH 8.0, 150 mM NaCl, 1% NP-40, 0.5% sodium deoxycholate, 0.1% SDS, protease inhibitor mixture and phosphatase inhibitor mixture (Sigma-Aldrich). Whole cell lysates, obtained from the supernatant after microcentrifugation at 14,000 rpm for 15 min at 4°C, were subjected to sodium dodecyl sulfate polyacrylamide gel electrophoresis (SDS-PAGE). The separated proteins were transferred to a nitrocellulose membrane and incubated with specific primary antibodies and horseradish peroxidase (HRP)-conjugated secondary antibodies. Antigens were visualized using an enhanced chemiluminescence substrate kit (Thermo Scientific).

**Quantitative real-time PCR:** RNA (2 μg) was converted to cDNA by reverse transcription using random hexamer primers, oligo dT and Reverse Transcription Master Premix (ELPIS Biotech, Daejeon, Korea). Quantitative real-time PCR analyses were performed using the cDNA from reverse transcription and gene-specific oligonucleotides in the presence of TOPreal qPCR 2X premix (Enzynomics, Daejeon, Korea). The PCR conditions were as follows: an initial denaturation step for 10 min at 95°C followed by 45 cycles of 95°C denaturation for 10 s, 58°C annealing for 15 s and 72°C elongation for 20 s. The melting curve of each PCR product was assessed for quality control. Supplementary Table 2 shows the sequences of the primers used for qPCR.

**Cardiolipin staining:** The cells were stained with 100 nM NAO for 30 min and 1 μg/ml of DAPI for 5 min. The stained cells were observed using an LSM700 confocal laser scanning microscope (Carl Zeiss, Oberkochen, Germany).

**Radioactive labeling and TLC:** Cardiolipin turnover was measured after labeling with [^32^P]P_i_ (20μCi/ml) for 24hr. New media was added after washing out [^32^P]P_i_ containing media at zero time, cells were harvested at the time indicated. Lipid extraction and TLC were performed as described previously ([Raemy *et al.* 2016](#_ENREF_1)). Image was obtained using phosphor imager and analyzed using Multi Gauge software (Fuji Film, Tokyo, Japan).

**Cell growth rate, colony-forming assay, and cell cycle analysis:** For analyzing the cell growth rate, cells were trypsinized and collected via microcentrifugation at 5,800 rpm for 5 min. Re-suspended cells were counted using a hemocytometer. The growth curve of MEFs is expressed by PDL, and the PDL is calculated by the following formula. PDL = X + 3.322 (log Y -log I) where, X = initial population doubling level, I = Initial cell number seeded into your vessel, Y = final cell Yield, or the number of cells at the end of the growth period. The colony-forming assay was performed using a Diff-Quick staining kit (Sysmex, Kobe, Japan). Cells were seeded at 1x10^4^ cells in 60-mm dishes, grown for 7 days and stained with a Diff-Quick staining kit. The stained colonies were counted. For cell cycle analysis, the collected cells were incubated in PBS containing 1 mg/ml RNase and 50 mg/ml propidium iodide (Sigma-Aldrich) for 30 min. The stained cells were subjected to FACS on a FACS Calibur flow cytometer (BD Bioscience, San Diego, CA, USA).

**Statistical Analysis:** Statistical values are presented as the mean ± S.D. A two-tailed Student’s *t*-test was used to compare between groups.

**Supplementary figure legends**

**Supplementary Figure 1. Caveolin-1 knockdown-induced senescence is the si-Cav-1 specific effect.**

A549 cells were treated with empty vector or RNAi-resistant Cav-1 vector (1^st^ si). After 24 hours, the A549 cells were further treated with si-CON or si-Cav-1 (2^nd^ si) (A-C). Quantification of relative cell number (A), β-Gal staining positivity (B) and immunoblotting (C) were determined 3 days after the second transfection. All data are shown as the mean ± S.D. Statistical significance was determined using Student's *t*-test. ^*^*p* < 0.05, ^**^*p* < 0.01 and ^***^*p* < 0.001.

**Supplementary Figure 2. Caveolin-1 knockdown induces premature senescence in various cell lines.**

HCT116 cells were treated with 100 nM si-CON or si-Cav-1 (A-C). Three days after siRNA treatment, the cell number was counted and presented as relative values (A). Five days after siRNA treatment, cellular morphology and β-gal staining positivity were observed. β-gal positivity was statistically quantified by calculating the ratio of stained cells to total cells (B). Cells were harvested at the indicated times after siRNA treatment and were subjected to immunoblotting analysis (C). HDFs were treated with 100 nM si-CON or si-Cav-1 (D-F). Three days after siRNA treatment, the cell number was counted and presented as relative values (D). Three days after siRNA treatment, cellular morphology and β-gal staining positivity were observed. β-gal positivity was statistically quantified by calculating the ratio of stained cells to total cells (E). Cells were harvested at the indicated times after siRNA treatment and were subjected to immunoblotting analysis (F). H460 cells were treated with 100 nM si-CON or si-Cav-1 (G-I). Three days after siRNA treatment, the cell number was counted and presented as relative values (G). Three days after siRNA treatment, cellular morphology and β-gal staining positivity were observed. β-gal positivity was statistically quantified by calculating the ratio of stained cells to total cells (H). Cells were harvested at the indicated times after siRNA treatment and were subjected to immunoblotting analysis (I). All data are shown as the mean ± S.D. Statistical significance was determined using Student's *t*-test. ^*^*p* < 0.05, ^**^*p* < 0.01 and ^***^*p* < 0.001.

**Supplementary Figure 3. Caveolin-1 knockdown leads to mitochondrial dysfunction.**

A549 cells were treated with 100 nM si-CON or si-Cav-1 for 24 h (A-E). The OCR (A) and ECAR (B) were measured using an XF24 analyzer and normalized to protein concentration. The intracellular ATP level was measured using a luminescent luciferase assay, and the ATP content was normalized to the protein amount. For a positive control, the cells were treated with 5 μg/ml oligomycin for 6 h (C). Intracellular ROS generation was analyzed by FACS after staining the cells with 5 μM MitoSox red for 15 min. For a positive control, the cells were treated with 0.5μg/ml doxorubicin for 12 h (D). Genomic DNA (RPS 18) and mitochondrial DNA (mtDNA, COX2) content were determined by qPCR. COX2 mtDNA content was normalized to RPS 18 genomic DNA (E). A549 cells were treated with 100 nM si-CON or si-Cav-1 for the indicated times. Whole cell lysates were immunoblotted for CI (NDUFV1 and V2 and NDUFS3), CII (SDHB), CIII (UQCRC2), CIV (subunit Vb) and CV (ATPβ) expression using actin as a loading control (F). A549 cells were treated with 100 nM si-CON or si-Cav-1 for 24 h. The cells were stained with 100 nM NAO for 30 min. Fluorescence intensity was measured using ZEN2009 software (G). Amount of [^32^P]P_i_ labeled cardiolipin in mitochondria of si-CON or si-Cav-1 treated A549 cells was measured using phosphor-imager after TLC (H). A549 cells were treated with 100nM si-CDS1 (I-K). Three days after siRNA treatment, the cell number was counted and presented as relative values (I). Four days after siRNA treatment, cellular morphology and β-gal staining positivity were observed. β-gal positivity was statistically quantified by calculating the ratio of stained cells to total cells (J). Three days after siRNA treatment cells were harvested and were subjected to immunoblotting analysis (K). All data are shown as the mean ± S.D. Statistical significance was determined using Student's *t*-test. ^*^*p* < 0.05, ^**^*p* < 0.01 and ^***^*p* < 0.001.

**Supplementary Figure 4. CI dysfunction induces premature senescence.**

A549 cells were treated with 100 nM si-CON or si-NDUFV1 (A-C). Three days after siRNA treatment, the cells were counted and presented as relative values (A). Three days after siRNA treatment, cellular morphology and β-gal staining positivity were observed. β-gal positivity was statistically quantified by calculating the ratio of stained cells to total cells (B). Cells were harvested at the indicated times after siRNA treatment and were subjected to immunoblotting analysis (C). A549 cells were treated with 100 nM si-CON or si-NDUFV1 for 24 h (D-H). The OCR (D) and ECAR (E) were measured using an XF24 analyzer and were normalized to the protein concentration. The intracellular ATP level was measured using a luminescent luciferase assay, and the ATP content was normalized to the protein amount. As a positive control, the cells were treated with 5 μg/ml oligomycin for 6 h (F). The intracellular ROS generation was analyzed by FACS after staining the cells with 5 μM MitoSox red for 15 min. For a positive control, the cells were treated with 0.5μg/ml doxorubicin for 12 h (G). Intracellular NAD^+^ and NADH levels were measured using a quantitative colorimetric assay kit (H). All data are shown as the mean ± S.D. Statistical significance was determined using Student's *t*-test. ^*^*p* < 0.05, ^**^*p* < 0.01 and ^***^*p* < 0.001.

**Supplementary Figure 5. Cav-1 knockdown-induced senescence results from SIRT1 inactivation.**

A549 cells were treated with 100 nM si-CON or si-Cav-1 for 6 h and then treated with 100 μM NMN for 3 days (A-C). Relative cell number (A) and β-Gal staining positivity (B) were quantified, and immunoblotting was performed (C). A549 cells were treated with 100 nM si-CON or si-Cav-1 for 6 h and then treated with 100 mM pyruvate for 3 days (D-F). Relative cell number (D) and β-Gal staining positivity (E) were quantified, and immunoblotting was performed (F). All data are shown as the mean ± S.D. Statistical significance was determined using Student's *t*-test. ^*^*p* < 0.05, ^**^*p* < 0.01 and ^***^*p* < 0.001.

**Supplementary Figure 6. The mRNA and protein expression levels of enzymes in cardiolipin biosynthesis pathway.**

The mRNA expression levels of CDS2, CRLS, PTPMT1, TAZ and Cav-1 were determined by qPCR in Cav-1^+/+^ and Cav-1^-/-^ MEFs at passage number 3 (A). A549 cells were treated with 100 nM si-CON or si-Cav-1 for 24 h (B-C). qPCR (B) and immunoblotting (C) was performed. All data are shown as the mean ± S.D. Statistical significance was determined using Student's *t*-test. ^*^*p* < 0.05, ^**^*p* < 0.01 and ^***^*p* < 0.001.

**Supplementary Figure 7. Cav-1 knockdown prevents tumor growth in a xenograft mouse model.**

H460 cells (5 x 10^6^) were subcutaneously injected into BALB/c athymic mice (A-D). Tumor size was measured for 13 days after the injection of either si-CON or si-Cav-1 on 7^th^ days. Tumor volume in the xenograft mice (n=6 or 5, respectively) was measured at the indicated times. The right panel shows photographs of the tumor-bearing mice (A). Tumor weight was determined after tumor isolation (B). The isolated tumors were photographed, and partial slices were stained with X-gal (C). The isolated tumors were assessed by immunoblotting for SIRT1, Ac-p53, p53, p21 and Cav-1 expression using actin as a loading control (D). All data are shown as the mean ± S.D. Statistical significance was determined using Student's *t*-test. ^*^*p* < 0.05, ^**^*p* < 0.01 and ^***^*p* < 0.001.
